# A cytokine/PTX3 prognostic index as a predictor of mortality in sepsis

**DOI:** 10.3389/fimmu.2022.979232

**Published:** 2022-09-15

**Authors:** Sadaf Davoudian, Daniele Piovani, Antonio Desai, Sarah N. Mapelli, Roberto Leone, Marina Sironi, Sonia Valentino, Rita Silva-Gomes, Matteo Stravalaci, Fatemeh Asgari, Alessandra Madera, Daniele Piccinini, Carlo Fedeli, Denise Comina, Stefanos Bonovas, Antonio Voza, Alberto Mantovani, Barbara Bottazzi

**Affiliations:** ^1^ Department of Research in Inflammation and Immunology, IRCCS Humanitas Research Hospital, Milan, Italy; ^2^ Department of Biomedical Science, Humanitas University, Milan, Italy; ^3^ Department of Emergency, IRCCS Humanitas Research Hospital, Milan, Italy; ^4^ The William Harvey Research Institute, Queen Mary University of London, London, United Kingdom

**Keywords:** sepsis, biomarkers, cytokines, PTX3, disease severity

## Abstract

**Background:**

Early prognostic stratification of patients with sepsis is a difficult clinical challenge. Aim of this study was to evaluate novel molecules in association with clinical parameters as predictors of 90-days mortality in patients admitted with sepsis at Humanitas Research Hospital.

**Methods:**

Plasma samples were collected from 178 patients, diagnosed based on Sepsis-3 criteria, at admission to the Emergency Department and after 5 days of hospitalization. Levels of pentraxin 3 (PTX3), soluble IL-1 type 2 receptor (sIL-1R2), and of a panel of pro- and anti-inflammatory cytokines were measured by ELISA. Cox proportional-hazard models were used to evaluate predictors of 90-days mortality.

**Results:**

Circulating levels of PTX3, sIL-1R2, IL-1β, IL-6, IL-8, IL-10, IL-18, IL-1ra, TNF-α increased significantly in sepsis patients on admission, with the highest levels measured in shock patients, and correlated with SOFA score (PTX3: r=0.44, p<0.0001; sIL-1R2: r=0.35, p<0.0001), as well as with 90-days mortality. After 5 days of hospitalization, PTX3 and cytokines, but not sIL-1R2 levels, decreased significantly, in parallel with a general improvement of clinical parameters. The combination of age, blood urea nitrogen, PTX3, IL-6 and IL-18, defined a prognostic index predicting 90-days mortality in Sepsis-3 patients and showing better apparent discrimination capacity than the SOFA score (AUC=0.863, 95% CI: 0.780−0.945 *vs.* AUC=0.727, 95% CI: 0.613-0.840; p=0.021 respectively).

**Conclusion:**

These data suggest that a prognostic index based on selected cytokines, PTX3 and clinical parameters, and hence easily adoptable in clinical practice, performs in predicting 90-days mortality better than SOFA. An independent validation is required.

## Introduction

Sepsis is a life-threatening condition due to a dysregulated response to infection which can lead to shock, multiple organ failure, and death. The estimated number of incident cases of sepsis increased to almost 50 million worldwide (1–4) and, despite massive efforts, the mortality rate has not improved over time. Early diagnosis of sepsis and prognostic assessment are crucial to prevent progression to severe disease and to make use of timely and appropriate treatments to reduce mortality (1–4).

Despite the recent revision of the definition of sepsis, diagnosis is still challenging for clinicians, in particular in the identification of patients at early stages (5). Alongside the diagnostic question, also important is the need to have early prognostic indications allowing clinicians to activate the most appropriate therapies based on the mortality risk of each individual patient (2, 6–10). A recent review collected 5367 studies identifying 258 different biomarkers of sepsis with potential diagnostic and/or prognostic functions (11). Among the different molecules, procalcitonin (PCT) and C-reactive protein (CRP) resulted as the most studied candidate biomarkers. However, the clinical implication and molecular involvement of these proteins still needs to be clarified and currently there is not a single molecule validated as the “gold standard” biomarker for sepsis (11). In particular the comprehensive analysis of the literature reported by Pierrakos et al. underlined the limited value of studies evaluating a single biomarker as a prognostic factor, given that mortality in septic patients is related to multiple pathophysiological processes. Therefore, investigation on multiple sepsis-related molecules is strongly suggested as a strategy to develop a validated prognostic model (12–18).

Here we focused on two proteins of the innate immune system, the long pentraxin 3 (PTX3) and the soluble form of the Interleukin-1 type 2 receptor (sIL-1R2). PTX3 is a distant relative of CRP (19, 20) and a key component of the innate immunity. The molecule is expressed by different cell types, at highest levels by phagocytes (monocytes/macrophages and myeloid dendritic cells), in response to primary pro-inflammatory signals, TLR engagement, microbial recognition and tissue damage (21–23). sIL-1R2 is generated by enzymatic cleavage of membrane-bound IL-1R2 and is released from neutrophils and macrophages in response to both pro- and anti-inflammatory signals, acting as a “decoy receptor” which negatively regulates the IL-1 signaling pathway (24–28).

Increased levels of PTX3 have been associated with infectious disorders, including sepsis and septic shock, tuberculosis, dengue and meningitis (29–33). In all these conditions, PTX3 plasma levels correlated with severity and had prognostic value. In sepsis, available data were mainly obtained in ICU patients and indicated that PTX3 is associated with disease severity, organ dysfunction and 28-days or 90-days mortality (29, 31, 34–39). Only few reports were obtained in patients enrolled in emergency rooms, such as the work by Uusitalo-Seppälä et al., underlining the role of PTX3 as a prognostic biomarker of 28-days mortality (40) in that setting.

Similarly, circulating levels of sIL-1R2 are documented to increase in many inflammatory disorders, correlating with disease severity (41–45). Early studies also reported increased levels of sIL-1R2 in small cohorts of sepsis patients or in experimental endotoxemia models (46–48). Elevated levels of sIL-1R2 were measured in patients with clinically defined sepsis (45, 49), and in a group of ICU patients with systemic infections categorized according with Sepsis-1 criteria (44). Despite the correlation with severity, contrasting results were reported in terms of prediction of mortality: Van Der Poll et al. showed higher levels of sIL-1R2 in non-survivors compared to survivors (45), while Muller et al. reported no difference of sIL-1R2 levels in relation to mortality (44).

Reflecting the co-occurrence of inflammation and immunosuppressive mechanisms, both pro- and anti-inflammatory cytokines increase concurrently in the plasma of septic patients. Tumor necrosis factor (TNF-α) and Interleukin 1 beta (IL-1β), the most important primary pro-inflammatory mediators, are the cytokines most extensively studied in sepsis patients (50–52). Other pro-inflammatory cytokines in sepsis response include IL-6, IL-8, IL-12, interferon (INF)-γ, granulocyte-colony stimulating factor (G-CSF), IL-12, IL-17 and IL-18 (50, 53, 54). Among the specific anti-inflammatory mediators enriched in sepsis, IL-1ra, IL-4 and IL-10 are the most studied (47, 53, 55, 56). So far, data showed a complex network of interactions between different cytokines in sepsis. While some patients are characterized by rapid production of both pro and anti-inflammatory cytokines (57, 58), other septic individuals release predominantly anti-inflammatory mediators or show reduction of both types of molecules (59–62). Therefore, the available literature does not provide conclusive results. Of note, the circulating levels of cytokines differ massively from subject to subject and even within the same patient during the evolution of the disease. Hence it might be relevant to screen a set of different cytokines for monitoring sepsis patients (63, 64).

Due to the complexity of sepsis, the measurement of a set of markers related to different pathways may be more beneficial than relying on a single biomarker (11, 16–18). Thus, the aim of this study was to evaluate the prognostic value of a selection of promising innate immunity molecules, namely PTX3 and sIL-1R2, in combination with a set of pro- and anti-inflammatory cytokines. Circulating biomarkers will be integrated with clinical parameters, in order to improve the early prognostic assessment of sepsis patients in ED setting.

## Materials and methods

### Ethics approval

This study complied with the provisions of the Declaration of Helsinki and was approved by the Institutional Review Board of Humanitas Research Hospital (Approval n° 820/18). Patients were enrolled only after the signature of a written informed consent. In the case the patient was unable to provide consent, this was obtained from their relatives. Confidentiality of patient data was preserved and no patient identifiers were used in the dataset.

### Study design

We conducted a single center prospective observational study enrolling patients with suspected sepsis admitted to the Emergency Department (ED) of Humanitas Research Hospital (from now on referred as Humanitas Hospital) between October 2017 and February 2020. Patients presenting at the Emergency Department were evaluated based on the Sepsis-3 criteria defined by the Third International Consensus Definitions for Sepsis and Septic Shock (65). Sepsis-3 criteria are based on the following recommendations: i) presence of an infection; ii) presence of organ dysfunction, represented by an increase in the Sequential [Sepsis-related] Organ Failure Assessment (SOFA) score of 2 points or more; iii) presence of at least two of the following clinical criteria, that together constitute a new bedside clinical score termed quick SOFA (qSOFA): altered mentation, a respiratory rate of 22/min or greater, and systolic blood pressure of 100 mm Hg or less. Septic shock is defined as a subset of sepsis patients clinically identified by a vasopressor requirement to maintain a mean arterial pressure of 65 mm Hg or greater, serum lactate levels greater than 2 mmol/L (>18 mg/dL) in the absence of hypovolemia or altered mentation (Glasgow coma scale score < 15). All non-sepsis patients had a proof of infectious disease.

**Figure 1:**
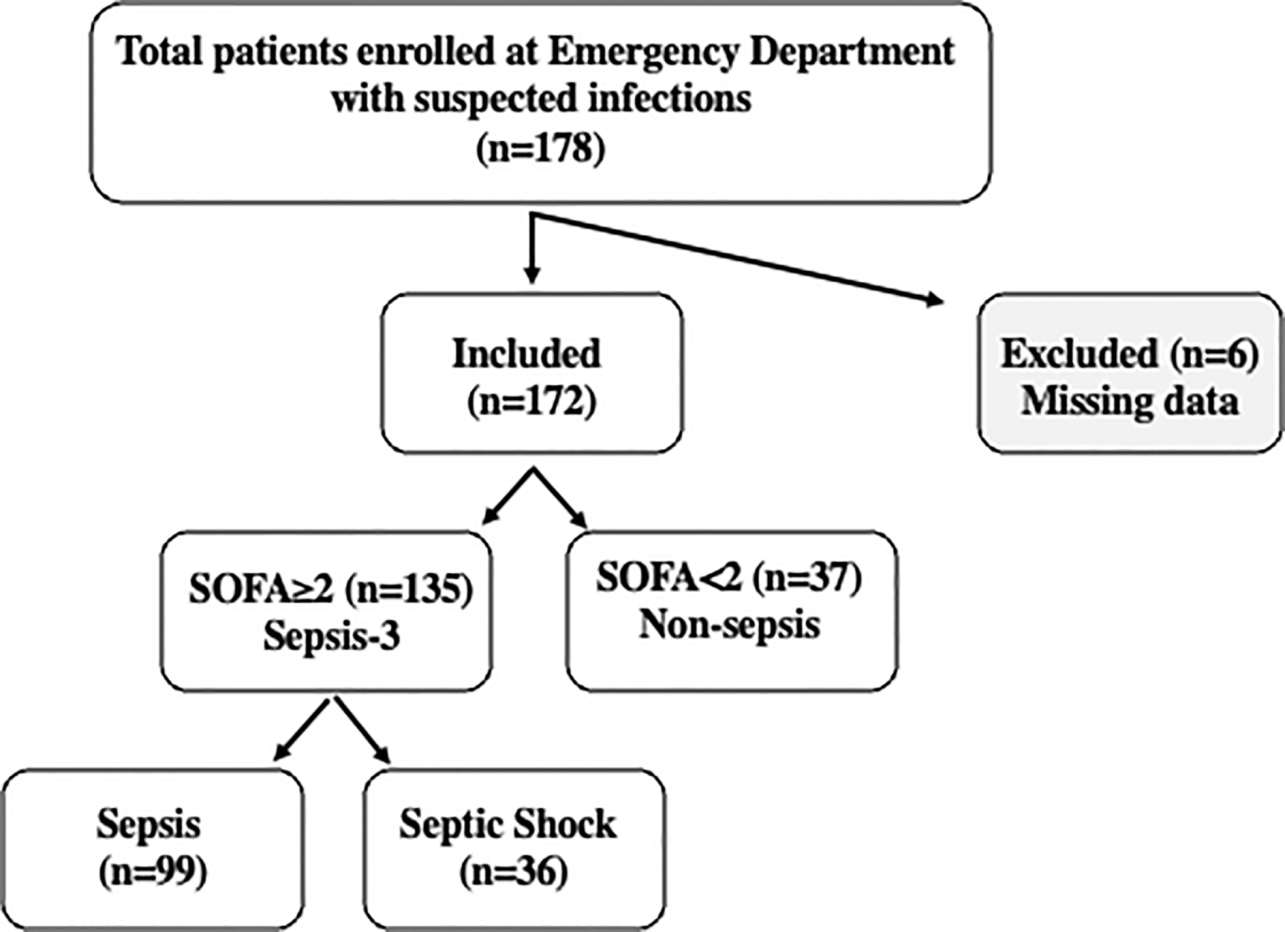
Flowchart of the study. A cohort of 178 patients was enrolled at the Emergency Department of Humanitas Research Hospital with suspected sepsis. 172 patients were included in the study and stratified based on Sepsis-3 criteria. Patients with SOFA<2 were classified as “non-sepsis” (n=37), while the 135 patients with SOFA‗2, were classified as “sepsis” (n=99) or “septic shock” (n=36) according to clinical evaluation. Septic shock patients were characterized by vasopressor requirement to maintain a mean arterial blood pressure of 65 mmHg or greater and serum lactate levels >2mm/L in the absence of hypovolemia or altered mentation (Glasgow coma scale score < 15).

A cohort of 178 patients aged 35-98 years old was enrolled in this study (**Figure 1**), representing approximately 25% of the individuals presenting at the ED of Humanitas Hospital with suspected sepsis during the recruitment period. Six patients were excluded due to missing data. The remaining population of 172 patients was stratified according to Sepsis-3 criteria as detailed above (65). Based on these criteria, the population was divided as follows: 37 patients with SOFA score < 2 were assigned to the “non-sepsis” group diagnosed with infections; 135 patients with SOFA≥2 were divided into two groups: sepsis, (n=99), and septic shock (n=36). The SOFA score was also used to determine the levels of organ dysfunction and mortality risk.

A group of healthy controls (HC) was enrolled among volunteers with age higher than 50 years (n=70). Median age and 25% and 75% quartiles (Q1–Q3) were 63 years (58–67); 56% were males and 43% were females.

### Sample collection and preparation

Blood samples were collected in EDTA tubes on the day of arrival at ED or when, within 24-72 hours of observation in ED, the patient was diagnosed as sepsis or septic shock. The day of first blood withdrawal was considered as day1. For those patients admitted to Humanitas Hospital, a second collection was done on day 5 ± 1. Blood samples were centrifuged at 1800 rpm for 10 minutes, then plasma was aliquoted and stored at -20°C until analysis. To ensure the reproducible quality of samples, blood tubes were processed within 2 hours after collection.

### Data collection

Demographic information (age, sex, prior medical history), ED stay, hospital stay, ICU admission and blood pressure were collected and documented. In addition, the progression of sepsis and survival outcome at 90 days were recorded. Routine laboratory tests were performed by methods applied in the Clinical Laboratory of Humanitas Hospital.

### Biomarker measurement

PTX3 and sIL-1R2 levels were measured using sandwich enzyme-linked immunosorbent assay (ELISA). PTX3 was measured by an in-house assay based on original reagents developed at Humanitas Hospital (detection limit 100 pg/ml; inter-assay and intra-assay variability ranges from 8 to 10%), as previously described (66). sIL-1R2 levels were measured using a commercial ELISA (Human sIL-1R2 Quantikine ELISA Kit, BioTechne; detection limit: less than 10 pg/ml) following the recommended protocol of the supplier. PCT and CRP concentrations were measured in the Clinical Laboratory of Humanitas Hospital.

A set of pro and anti-inflammatory cytokines, including IL-10, IL-6, IL-1β, TNF-α, IL-18, IL-8 and IL-1ra was measured using a customized assay in the ELLA Automated Immunoassay System (ProteinSimple, San Jose, CA, USA). The assay was performed following instructions provided by the manufacturer; concentrations of the different cytokines were directly provided by the software of the ELLA instrument. The lower limits of detection were: 0.46 pg/ml for IL-10; 0.41 pg/ml for IL-6; 0.16 pg/ml for IL-1β; 0.30 pg/ml for TNF-α; 0.96 pg/ml for IL-18; 0.19 pg/ml for IL-8; 3.39 pg/ml for IL-1ra.

### Statistical analysis

All statistical tests were two-sided. Stata 16.1 (StataCorp LLC, College Station, TX, USA), R software and GraphPad Prism 7.0 (GraphPad Software Inc., CA) were used for statistical analyses and graphics. All significant *P* values are reported in figures and tables legends.

Quantitative data were presented as means ± standard deviations (SD) or medians and interquartile ranges (Q1-Q3) according to normality (analyzed by the D’Agostino-Pearson test). Qualitative data were summarized as frequency (percentage). Parametric (Student’s *t*-test and one-way analysis of variance [ANOVA]) and non-parametric methods (two-sample Wilcoxon rank-sum [Mann-Whitney] test; Kruskal-Wallis (two-sample Wilcoxon rank-sum [Mann-Whitney] test; Kruskal-Wallis [equality-of-populations] rank test; Pearson’s chi-squared; and Fisher’s exact test) were used to detect differences between groups. Correlations were assessed by the Spearman’s rank correlation coefficient and the respective *p*-value. For hypothesis testing, a probability level lower than 0.05 was considered as statistically significant.

#### Multivariable model and prognostic index development

The outcome considered for model development was 90-days mortality. We used time-to-event (survival) methods for censored observations. Time to event was defined as the time from the baseline visit (admission to emergency department) until the date of event or censoring. Kaplan–Meier estimates were used to draw cumulative incidence curves, compared by log-rank tests, as well as by univariable Cox proportional hazards (PH) analysis.

Candidate predictors for the multivariable model included the following variables at baseline: age, gender, SOFA and qSOFA scores, white blood cells, lymphocytes, neutrophils, platelets, C-reactive protein, capillary refill time, hemoglobin, hematocrit, sodium, vitamin K, fasting blood sugars, prothrombin time, partial thromboplastin time, body temperature, heart rate, systolic and diastolic blood pressure, blood urea nitrogen, paO_2_, spO_2_, ratio of paO_2_/fiO_2_, biomarkers of inflammation (pentraxin-3, soluble IL-1R2, IL-1β, IL-1ra, IL-6, IL-8, IL-10, IL-18, TNFα), presence of cardiovascular disease, hypertension, malignancy, neurological disorders, diabetes type 2, chronic obstructive pulmonary disease, chronic kidney disease and other comorbidities. As the distribution of inflammatory biomarkers levels was severely positively skewed, we applied a log10-transformation prior to any analysis.

To avoid extreme collinearity, we first inspected pair-wise correlations among continuous predictors. In a few cases (e.g. hemoglobin and hematocrit) variables showed substantial collinearity (ρ-Spearman ≥0.80). In these cases, the variable most associated with mortality (highest likelihood ratio chi-squared test) was further considered for multivariable modelling. To assess associations between each candidate predictor and 90-days survival, we conducted explorative univariable Cox PH analysis. Except for the biomarkers of inflammation, candidate predictors showing a p-value <0.20 were considered for multivariable modelling. Given that the focus of the study was an in-depth screening of biomarkers of inflammation as potential predictors of mortality in septic patients, we explored systematically every potential interaction, also considering non-linearity through fractional polynomial terms (67). Potential interactions were screened across the other predictors at the final stage of model selection. The best fitting model was initially selected according to the lowest Akaike information criterion (68). Given the high number of candidate predictors relative to the limited sample size and number of events, we further simplified the resulting model through a backward selection procedure and eliminated variables not significant at a strict significance level of p<0.005. This process was deemed necessary to ensure a parsimonious multivariable model and avoid substantial overfitting of the data. The proportionality of hazard assumption was tested based on Shoenfeld residuals. Model fit was assessed through the Groennesby and Borgan test (69).

We assessed model discrimination using the Harrell’s c-statistic, which quantifies the ability to identify correctly those patients who will die over the study period (68). Each variable entering the final multivariable model was also assessed in term of individual discrimination capacity (continuous), and sensitivity and specificity (dichotomous) in predicting mortality at 90 days of follow-up. The optimal cut-point for each predictor was determined through the maximally selected rank statistics, providing a threshold value corresponding to the most significant association with mortality (70). To estimate sensitivity and specificity at 90 days of follow-up, we used time-dependent receiver operating characteristic (ROC) curve analysis by means of inverse probability of censoring weighting (71). In time-dependent ROC-curve analysis, the status of an individual is observed and updated at each time point taking into account censored observations.

A preliminary prognostic index was built by multiplying the multivariable model beta coefficients (including interactions) by each patient’s characteristics (age, blood urea nitrogen, and the log10-transformed PTX3, IL-6 and IL-18 levels) (68). Internal calibration was evaluated by plotting the observed proportion *vs.* predicted survival probability and reporting the calibration slope (which should equal one for a perfectly calibrated model) (68). We also performed a test for calibration intercept equals 0 and slope equals 1, as appropriate (68). The area under the curve of our preliminary prognostic index at 90-days were formally compared with those of SOFA and qSOFA scores, which represent the current gold standards for prediction of mortality in septic patients in different clinical settings.

A nomogram plot was produced to transform all possible total point scores into individual risks of death. We calculated the optimal cut-point of the continuous index by maximally selected rank statistics. This allowed estimating the sensitivity and specificity of this classification rule (i.e. patients scoring less points than the threshold are classified as “alive”, those scoring more or equal to the threshold are classified as “dead”) in predicting the 90-days risk of death.

## Results

### Patient demographics

**Figure 1** reports the flowchart of the population analyzed in this study: 178 patients with suspected sepsis were enrolled during admission to the ED of Humanitas Hospital. Six patients were excluded due to missing data. Following evaluation by clinicians, the remaining population of 172 patients included 135 patients with SOFA≥ 2, 99 patients with sepsis and 36 with septic shock, while 37 patients with SOFA< 2 did not meet the Sepsis-3 criteria, despite the presence of infections. These patients were categorized as “non-sepsis”.

**Table 1** summarizes the clinical characteristics of the overall population. The mean age was significantly increased with the severity, while no differences between males and females in the three subgroups of patients were observed. Most frequent infections were at the urinary tract (24% in non-sepsis and 41% in septic shock groups) and the respiratory tract (44% in sepsis group). Almost all the patients with sepsis and septic shock had at least one comorbidity. The most frequent comorbidity in non-sepsis and sepsis groups is represented by hypertension (40% and 34% respectively), while cardiovascular disease was the most frequent comorbidity in septic shock patients (50%). Among the laboratory and clinical results, SOFA score and levels of lactate, PCT, creatinine, d-dimer, potassium and urea were higher in septic shock patients than in the other groups of patients. Also, vasopressor need and hypotension increased significantly in septic shock patients.

**Table 1:** Demographic, laboratory and clinical characteristics of the entire patients population on day 1.

Variables	Non-sepsis (n=37)	Sepsis (n=99)	Septic shock (n=36)	*p* value
**Demographic Characteristics**				
**Age, median [Q1-Q3]**	70 [57-81]	78 [70-84]	78 [71-85]	**0.02**
**Male sex, n (%)**	21 (56.8)	68 (68.7)	21 (58.3)	0.32
**Female sex, n (%)**	16 (43.2)	31 (31.3)	15 (41.7)	0.27
**Comorbidities n (%)**				
**Cardiovascular disease**	10 (27.0%)	33 (33.3%)	18 (50.0%)	0.06
**Hypertension**	15 (40.5%)	34 (34.3%)	12 (33.3%)	0.76
**Malignancy**	13 (35.1%)	31 (31.3%)	14 (38.8%)	0.69
**Neurological disease**	9 (24.3%)	22 (22.2%)	7 (19.4%)	0.88
**Diabetes type 2**	5 (13.5%)	19 (19.1%)	6 (16.6%)	0.73
**COPD**	6 (16.2%)	17 (17.1%)	10 (27.7%)	0.33
**Chronic kidney disease**	1 (2.7%)	19 (19.1%)	5 (13.8%)	**0.01**
**Others**	9 (24.3%)	27 (27.2%)	8 (22.2%)	0.97
**Site of infection n (%)**				
**Respiratory system**	7 (18.9%)	44 (44.4%)	9 (25.0%)	**0.008**
**Urinary tract**	9 (24.3%)	29 (29.2%)	15 (41.6%)	0.24
**Liver**	5 (13.5%)	4 (4.0%)	4 (11.1%)	0.11
**Abdomen**	2 (5.4%)	4 (4.0%)	0 (0%)	0.40
**Blood**	1 (2.7%)	4 (4.0%)	2 (5.5%)	0.82
**Skin and soft tissue**	3 (8.1%)	4 (4.0%)	1 (2.7%)	0.50
**Unknown**	4 (10.8%)	6 (6.0%)	3 (8.3%)	0.63
**Others**	6 (16.2%)	4 (4.0%)	2 (5.5%)	0.04
**Vital signs, median [Q1-Q3]**				
**Body temperature (°C)**	37.6 [36.7-38.4]	37.3 [36.3-38.4]	36.8 [36.0-38.5]	0.67
**Body mass index (kg/m²)**	26.8 [24.2-32.3]	26.3 [22.3-31.3]	24.5 [21.1-25.6]	0.25
**Respiratory rate (per min)**	16 [15-18]	18 [16-21]	18 [16-20]	**0.03**
**Heart rate (bpm)**	97.5 [85.5-109.5]	97 [78-110]	99 [82.5-119.5]	0.70
**SBP (mmHg)**	116 [103-130]	110 [95-134]	83 [76-92]	**0.0001**
**DBP (mmHg)**	65 [60-79]	65 [50-80]	51 [40-60]	**0.0001**
**MAP (mmHg)**	85 [77-93]	77 [67-90]	60 [53-65]	**0.0001**
**GCS**	15 [15-15]	15 [15-15]	15 [13-15]	0.06
**Laboratory values, median [Q1-Q3]**				
**WBC (10^3/mm^3)**	11.9 [8.3-16.6]	11.6 [8.4-18.8]	16.1 [7.6-20.0]	0.46
**Lymphocytes (10^3/mm^3)**	0.8 [0.5-1.2]	0.7 [0.4-1.1]	0.7 [0.4-1.0]	0.30
**Neutrophils (10^3/mm^3)**	9.8 [6.7-13.9]	9.2 [6.8-16.4]	14.3 [6.6-17.5]	0.29
**Platelets (10^3/mm^3)**	204 [170-301]	172 [123-270]	166 [117-272]	0.10
**Lactate (mmol/L)**	1.6 [1.1-3.0]	2.6 [1.6-4.0]	4.8 [2.7-7.1]	**0.0002**
**INR (sec)**	1.2 [1.1-1.4]	1.3 [1.1-1.5]	1.3 [1.2-1.7]	0.09
**Creatinine (mg/dL)**	0.8 [0.6-1.0]	1.6 [1.0-2.5]	1.9 [1.2-3.2]	**0.0001**
**D-dimer (ng/ml)**	743 [416-2485]	685 [383-1896]	1728 [787-4735]	**0.01**
**Fibrinogen (mg/dL)**	633 [459-798]	512 [414-714]	504 [386-665]	0.39
**Hemoglobin (g/dl)**	12.0 [10.4-12.7]	11.6 [10.5-13.1]	10.5 [9.0-12.4]	0.12
**Hematocrit (%)**	36.6 [31.2-37.7]	35.6 [32.0-39.8]	34.3 [27.9-38.5]	0.30
**Bilirubin (mg/dL)**	0.7 [0.5-1.0]	0.8 [0.6-1.1]	0.9 [0.6-1.4]	0.15
**Sodium (mmol/L)**	137 [134-139]	137 [133-140]	138 [135-141]	0.56
**Potassium (mmol/L)**	3.8 [3.5-4.1]	4.0 [3.6-4.5]	4.3 [3.7-5.1]	**0.009**
**Urea (mg/dL)**	39.0 [31.0-59.7]	74.3 [49.9-115.3]	89.5 [52.8-148.4]	**0.0001**
**Fasting blood sugar (mg/dL)**	124 [106-165]	124 [105-169]	109 [85-184]	0.22
**PT (second)**	1.2 [1.2-1.4]	1.3 [1.1-1.5]	1.3 [1.2-1.6]	0.28
**PTT (second)**	1.0 [0.9-1.0]	0.9 [0.9-1.1]	1.0 [0.8-1.1]	0.46
**Bicarbonate (mmol/L)**	24.4 [24.0-26.3]	22.6 [20.0-27.1]	19.1 [16.7-23.4]	**0.04**
**PH value**	7.5 [7.4-7.5]	7.5 [7.4-7.5]	7.4 [7.4-7.5]	0.18
**PCT (ng/ml)**	0.4 [0.1-1.3]	3.6 [0.6-17.5]	14.6 [2.3-75.5]	**0.0001**
**CRP (mg/dL)**	9.7 [3.6-19.6]	15.4 [6.7-24.1]	16.6 [12.2-22.4]	0.10
**PTX3 (ng/ml)**	34.6 [11.6-68.2]	52.5 [22.4-177.0]	210.9 [103.8-613.8]	**0.0001**
**sIL-1R2 (ng/ml)**	17.1 [14.6-20.0]	23.5 [16.1-34.6]	31.0 [22.0-50.1]	**0.0001**
**Clinical factors, median [Q1-Q3] or n (%)**				
**SOFA score**	1 [0-1]	4 [3-6]	8 [5-9]	**0.0001**
**qSOFA score**	0 [0-0]	1 [0-1]	1.5 [1-2]	**0.0001**
**ICU admission**	0 (0)	3 (3.0)	5 (17.2)	0.39
**Vasopressor need**	0 (0)	2 (2.0)	22 (61.1)	**0.0001**
**Hypotension**	1 (2.7)	26 (26.3)	32 (88.9)	**0.0001**
**Hospital stays (days)**	11 [7-17]	10 [7-18]	15 [4-24]	0.76

Data are reported as median [Q1-Q3] or n (%). Kruskal-Wallis equality-of-populations rank test was used for the comparisons of the different variables across the three groups of patients. Statistically significant values are in bold character. Abbreviations: COPD, Chronic obstructive pulmonary disease; DBP, diastolic blood pressure; GCS, Glasgow coma scale; ICU, intensive care unit; INR, international normalized ratio; MAP, mean arterial pressure; PCT, procalcitonin; PT, prothrombin time; PTT, partial thromboplastin time; SBP, systolic blood pressure; SOFA, Sequential Organ Failure Assessment; qSOFA, quick Sequential Organ Failure Assessment; WBC, white blood cells.

### PTX3 and sIL-1R2 levels in sepsis and association with severity

**Figure 2:**
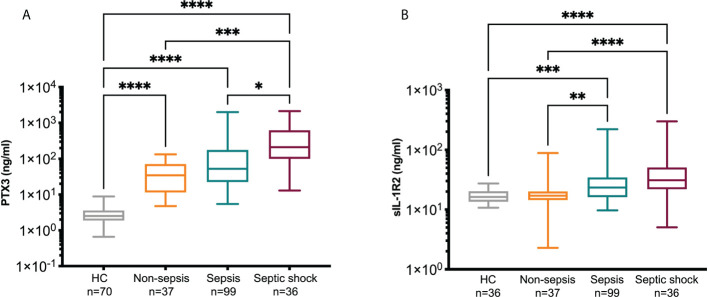
PTX3 and sIL-1R2 levels on day 1. PTX3 **(A)** and sIL-1R2 **(B)** plasma concentration were measured in non-sepsis, sepsis and septic shock patients admitted to ED. A group of healthy controls (HC) was also included. Kruskal-Wallis equality-of-populations rank test was used for the comparisons, * *p*≤ 0.05; **p≤ 0.01; ***p≤ 0.001; ****p≤ 0.0001.

The levels of PTX3 and sIL-1R2 in HC and in non-sepsis, sepsis and septic shock patients on day 1 of ED admission are reported in **Figure 2** and **Table 1**. PTX3 levels were significantly increased in all the three groups of patients compared to HC (**Figure 2A**; PTX3 levels in HC: 2.52 [1.88-3.6], median ng/ml [Q1-Q3]). In the same cohort, sIL-1R2 levels were also increased in sepsis and septic shock patients compared to HC (median and Q1-Q3] 16.29 ng/ml [13.52-20.25]), while normal levels of sIL-1R2 were observed in non-sepsis patients (**Figure 2B**). The increased severity among the three groups of patients is supported by the upregulated levels of several clinical parameters, which are indicative of sepsis severity (**Table 1**). In parallel, both PTX3 and sIL-1R2 are increased with the increasing of severity from non-sepsis to sepsis and septic shock (**Figure 2** and **Table 1**).

For the patients admitted to wards we collected a second blood sample after 5 days of hospitalization (non-sepsis n=23, sepsis n=62, septic shock n=17). In most of the patients with matched samples at day 1 and 5 we observed a significant decrease of PTX3 after 5 days of hospitalization, while sIL-1R2 levels did not change (**Table 2**). PCT and CRP, two molecules widely used as biomarkers indicative of sepsis severity (72, 73), were also elevated in our cohort of patients compared to levels in healthy population [CRP<0.5 mg/dl; PCT 0.05-0.5 ng/ml (74–76)], and were higher in Sepsis-3 patients compared to non-sepsis individuals (CRP: *p*=0.04; PCT: *p*<0.0001, Mann-Whitney test). In addition, a decrease was observed from day 1 to day 5 (not shown).

**Table 2:** PTX3 and sIL-1R2 levels at day 1 and day 5 in non-sepsis, sepsis and septic shock patients.

	PTX3 Day 1	PTX3 Day 5	p value	sIL-1R2 Day 1	sIL-1R2 Day 5	p value
**Non-sepsis** **(n=23)**	48.9 [28.1-82.5]	16.6 [10.0-30.7]	**0.001**	17.0 [12.0-20.0]	18.1 [13.1-21.1]	0.66
**Sepsis** **(n=62)**	69.4 [34.1-220.6]	17.6 [10.2-28.9]	**<0.0001**	23.9 [17.9-35.1]	25.0 [17.28-35.6]	0.69
**Septic shock** **(n=17)**	219.8 [69.9-832.0]	30.31 [10.0-48.7]	**0.0001**	34.37 [21.3-72.2]	41.6 [15.9-82.4]	0.54

Data are reported as median ng/ml and [Q1-Q3], and refer only to those patients with available samples for both the time points. Number of patients is indicated for each group. The Wilcoxon signed-ranks test was used for the comparisons of PTX3 and sIL-1R2 levels on day 1 and day 5 in the different groups of patients. Statistically significant values are in bold character.

Univariable Spearman’s rank correlation analysis of PTX3 and sIL-1R2 with SOFA score showed a positive correlation (PTX3: r=0.44, *p*<0.0001; sIL-1R2: r=0.35, *p*<0.0001, **Table S1**). In addition, PTX3 and sIL-1R2 correlated with different clinical parameters evaluated at enrollment in ED (e.g. creatinine and D-dimer, **Table S1**) as well as with PCT and CRP. Finally, a correlation was observed between PTX3 and sIL-1R2 evaluated on day 1.

### Cytokine levels in Sepsis-3 population

In the same samples we measured a panel of pro-inflammatory (IL-6, IL-1β, TNF-α, IL-18 and IL-8) and anti-inflammatory cytokines (IL-10, IL-1ra). On day 1 all the cytokines were highly increased in patients compared to HC, with a gradual trend of progressively higher levels from non-sepsis to sepsis and septic shock patients (**Table 3**). In particular, there was approximately a 2-fold increase of all cytokines except IL-18 in septic shock patients compared to sepsis patients on day 1. When we limited the analysis to those patients admitted to wards, circulating levels of all cytokines decreased from day 1 to day 5, with the only exception of IL-1β in septic shock patients and IL-18 in sepsis patients (**Table S2**).

**Table 3:** Cytokine levels in healthy controls, non-sepsis, sepsis and septic shock patients on day 1 of admission to Emergency Department.

Cytokine	Healthy control (n=20)	Non-sepsis (n=37)	Sepsis (n=99)	Septic shock (n=36)	*p* value
**IL-10**	1.0 [0.7-1.4]	11.4 [5.8-29.0]	16.3 [8.3-41.0]	46.5 [15.2-333.5]	**0.0001**
**IL-1β**	0.1 [0.0-0.3]	0.8 [0.5-1.4]	0.8 [0.5-1.3]	1.3 [0.7-2.9]	**0.0001**
**IL-6**	1.6 [1.0-2.9]	82.0 [30.6-128.2]	112.9 [35.2-230.3]	342.7 [125.7-8450.4]	**0.0001**
**TNF-α**	4.9 [4.2-6.0]	14.9 [11.1-20.8]	23.1 [14.0-37.1]	45.5 [21.4-164.7]	**0.0001**
**IL-18**	138.9 [87.9-203.5]	262.6 [201.7-387.8]	328.8 [226.9-525.7]	376.3 [256.6-625.2]	**0.0001**
**IL-1ra**	183.5 [141.4-259.9]	2218.8 [1206.0-3258.2]	4005.4 [1418.5-9393.3]	15527.6 [4123.4-46700.6]	**0.0001**
**IL-8**	5.2 [4.1-8.5]	16.1 [10.9-27.5]	29.6 [15.1-73.0]	74.7 [42.2-352.3]	**0.0001**

Data are reported as median pg/ml and [Q1-Q3]; Kruskal-Wallis equality-of-population test was used for the comparison of cytokines levels among the non-sepsis, sepsis and septic shock groups of patients. Number of subjects with available data is indicated for each group. Statistically significant values are in bold character.

PTX3 and sIL-1R2 showed a positive correlation with all the pro- and anti-inflammatory cytokines considered in the present analysis, except IL-18 and IL-1β (**Table 4**). The strongest correlations were observed between the C-X-C chemokine IL-8, a main chemotactic factor for neutrophils, and PTX3 (r=0.62, *p*<0.0001) or sIL-1R2 (r=0.50, *p*<0.0001) respectively. In addition, both pro and anti-inflammatory cytokines were positively correlated with SOFA score on day 1 of admission. Among them, TNF-α showed the strongest correlation (r=0.60, *p*<0.0001, **Table 4**).

**Table 4:** Univariable correlations of PTX3, sIL-1R2 and SOFA score with cytokines in the Sepsis-3 population (n=135).

	PTX3		sIL-1R2		SOFA score	
Cytokine	Spearman r	*p*	Spearman r	*p*	Spearman r	*p*
**IL-10**	0.50	**<0.0001**	0.43	**<0.0001**	0.43	**<0.0001**
**IL-1β**	0.20	**0.01**	0.09	0.27	0.31	**0.0002**
**IL-6**	0.51	**<0.0001**	0.41	**<0.0001**	0.44	**<0.0001**
**TNF-α**	0.49	**<0.0001**	0.35	**<0.0001**	0.60	**<0.0001**
**IL-18**	0.12	0.13	0.31	**0.0002**	0.25	**0.0036**
**IL-1ra**	0.56	**<0.0001**	0.42	**<0.0001**	0.53	**<0.0001**
**IL-8**	0.62	**<0.0001**	0.50	**<0.0001**	0.58	**<0.0001**

Spearman’s rank correlation test was used. Statistically significant values are in bold character.

### Distribution of PTX3, sIL-1R2 and cytokines between survivors and non-survivors in Sepsis-3 patients

The 90-days survival analysis in the total Sepsis-3 population showed 99 survivors (73%) and 36 non-survivors (27%). Mortality rate was 20% among sepsis patients and 44% among septic shock patients. Of note, among the non-sepsis patients, four subjects (11%) did not survive following the 90-days follow up.

Clinical data, PTX3, sIL-1R2 and cytokines levels among survivors and non-survivors are summarized in **Table 5**. SOFA score, lactate, CRP, creatinine, d-dimer and urea on day 1 were remarkably higher in non-survivors compared to survivors. Similarly, PTX3 and sIL-1R2 levels at enrollment were significantly higher in patients who died within 90 days from ED admission compared to those who survived. In addition, both pro- and anti-inflammatory cytokines levels were upregulated in non-survivors. Additional data are reported in **Table S3**.

**Table 5:** Distribution of selected clinical parameters and biomarkers among survivors and non survivors in the Sepsis-3 population.

Variables	Survivors (n=99)	n	Non-Survivors (n=36)	n	*p* value
**SOFA score**	4 [3-6]	99	6.5 [4-8.5]	36	**0.0005**
**qSOFA score**	1 [0-1]	99	1 [1-2]	36	**0.001**
**Neutrophils (10^3/mm^3)**	9.5 [6.1-15.8]	98	14.1 [7.9-18.8]	36	**0.05**
**Lactate (mmol/L)**	2.7 [1.6-4.4]	57	4.5 [3.1-6.9]	21	**0.007**
**Creatinine (mg/dL)**	1.5 [1.1-2.3]	99	2.3 [1.4-4.5]	36	**0.01**
**D-dimer (ng/ml)**	685 [398-1821]	51	2843 [1256-4057]	21	**0.003**
**Urea (mg/dL)**	69.4 [49.9-105.7]	98	122.8 [63.4-177.6]	36	**0.002**
**Body temperature (°C)**	37.7 [36.4-38.5]	97	36.5 [36.0-38.0]	35	**0.01**
**MAP (mmHg)**	73 [62-88]	99	66 [55-77]	35	**0.02**
**DBP (mmHg)**	60 [50-75]	99	52 [44-70]	36	**0.03**
**GCS**	15 [15-15]	99	15 [13-15]	36	**0.003**
**FiO2 (%)**	21 [21-28]	99	21 [21-36]	36	**0.01**
**Haemoglobin (g/dl)**	11.7 [10.5-13.1]	99	10.6 [9.4-12.4]	36	**0.03**
**CRP (mg/dL)**	15.4 [6.6-21.9]	98	17.4 [13.1-26.9]	36	**0.01**
**PTX3 (ng/ml)**	59.9 [19.9-177.0]	99	237.0 [61.1-557.0]	36	**0.0001**
**sIL-1R2 (ng/ml)**	22.8 [15.8-33.8]	99	34.6 [24.9-50.9]	36	**0.0002**
**IL-10 (pg/ml)**	15.5 [8.6-32.8]	99	48.0 [16.8-137.2]	36	**0.0004**
**IL-1β (pg/ml)**	0.8 [0.5-1.4]	99	1.2 [0.7-2.0]	36	**0.01**
**IL-6 (pg/ml)**	113.8 [41.1-297.7]	99	239.3 [92.8-730.5]	36	**0.03**
**TNF-α (pg/ml)**	25.2 [15.6-42.2]	99	36.1 [19.0-82.7]	36	**0.05**
**IL-18 (pg/ml)**	307 [222.4-472.8]	99	418.3 [284.6-684.4]	36	**0.05**
**IL-1ra (pg/ml)**	4099.7 [1595.1-10281.7]	99	9569.6 [3668.2-34912.4]	36	**0.01**
**IL-8 (pg/ml)**	29.6 [16.0-73.4]	99	73.8 [34.7-204.4]	36	**0.0003**

Data are reported as median and [Q1-Q3]; Mann-Whitney test was used for the comparisons. Only variables statistically significant are reported in the table. Abbreviations: DBP, diastolic blood pressure; FiO2, fraction of inspired oxygen; GCS, Glasgow coma scale; MAP, mean arterial pressure; SOFA, Sequential Organ Failure Assessment; qSOFA, quick Sequential Organ Failure Assessment. Statistically significant values are in bold character.

### Multivariable model development

**Figure 3:**
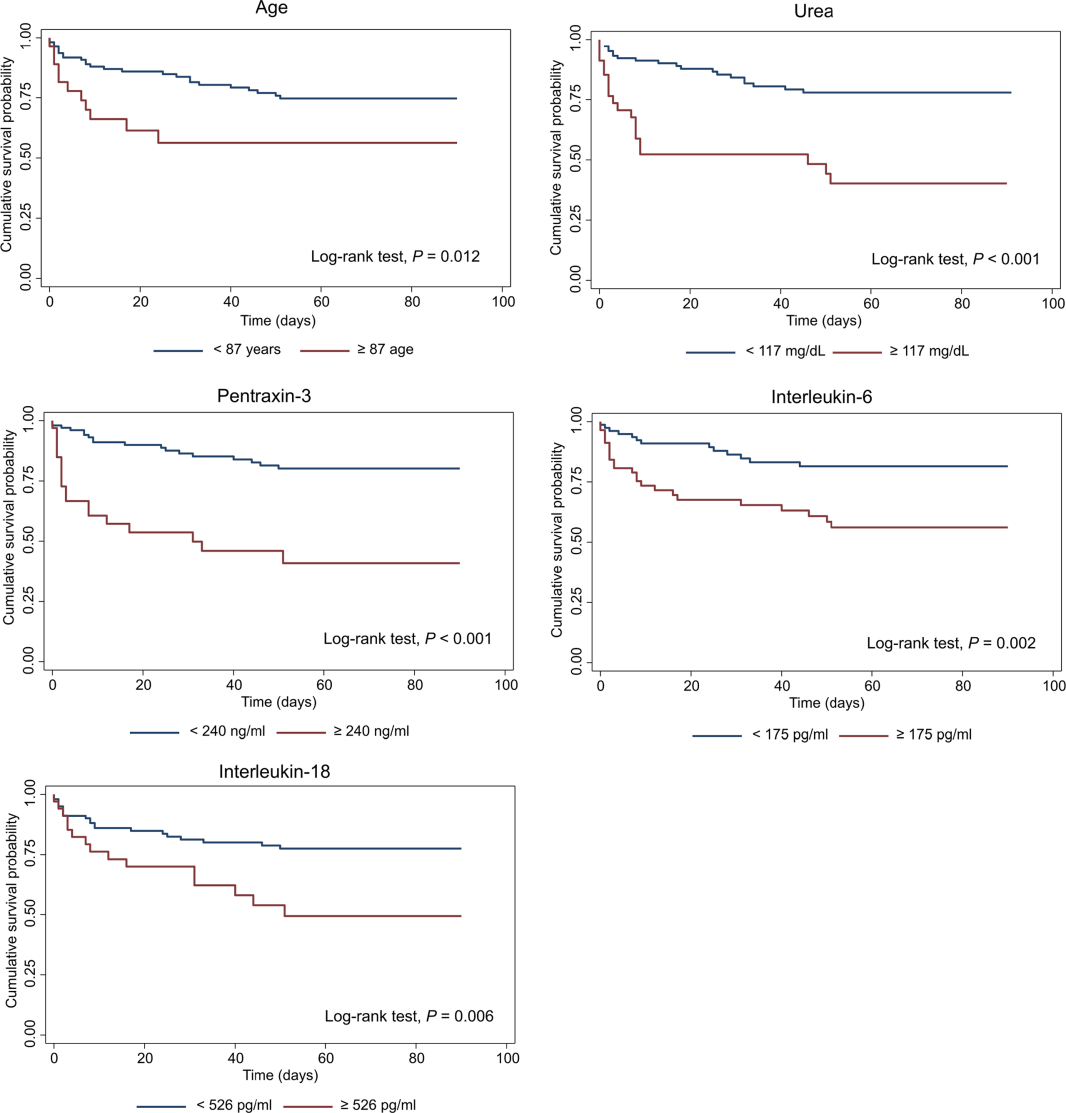
Kaplan-Meier survival curves of predictors included in the multivariable Cox model of 90-days mortality. Patients were categorized in two groups in accordance to continuous predictors dichotomized based on the maximally selected rank statistic. PTX3, IL-6 and IL-18 were log10-transformed before analysis. For each predictor, survival was analyzed by Log-rank test and correspondent *p* values are reported on the graphs.

The association between candidate predictors measured at the arrival at the ED, and 90-days mortality was first explored by univariable Cox proportional analysis. Variables not associated with mortality in this cohort of patients are reported in **Table S4**, while predictors with a significant hazard ratio are listed in **Table 6**. According to the analysis, PTX3 and sIL-1R2 emerged as strong predictors of mortality, with a hazard ratio respectively of 3.09 [1.80-5.30] and 5.31 [2.05-13.74] and *p*≤0.001 (**Table 6**). Combinations including the different biomarkers and clinical variables were then investigated. The most parsimonious multivariable Cox proportional hazard model comprising independent predictors of 90-days mortality included five variables: age, blood urea nitrogen, PTX3, IL-6 and IL-18 as continuous predictors. The three biomarkers of inflammation were log10-transformed. The model included a strong positive interaction (synergistic effect) between IL-6 and IL-18 (i.e. due to this strong interaction term, despite their negative coefficients, IL-6 and IL-18 should be interpreted as positively associated with 90-days mortality, though in a non-linear fashion). All variables were significant at the conservative threshold of *p*<0.005. For each predictor we calculated a threshold from the maximally selected rank statistics. The biomarkers threshold included: age (87 years), blood urea nitrogen (117 mg/dL), PTX3 (240 ng/ml), IL-6 (175 pg/ml), IL-18 (526 pg/ml). According to these thresholds, patients were categorized in two groups (i.e. high *vs.* low). In **Figure 3** are reported the Kaplan-Meier curves for each predictor included in the final multivariable model.

**Table 6:** Univariable Cox proportional hazard analysis of candidate predictors of 90-days mortality.

Variable (n)	Hazard ratio	95% CI	*p* value
**SOFA score (n=135)**	1.24	1.11−1.38	**<0.001**
**qSOFA score (n=135)**	2.03	1.37−3.02	**<0.001**
**Sodium (n=135)**	1.04	1.01−1.08	**0.011**
**Blood urea nitrogen (n=134)**	1.00	1.00−1.01	**0.001**
**PaO_2_/FiO_2_ (n=135)**	0.65	0.45−0.95	**0.026**
**Biomarkers (n=135)**			
**PTX3 (n=135)**	3.09	1.80−5.30	**<0.001**
**sIL-1R2 (n=135)**	5.30	2.05−13.74	**0.001**
**CRP (n=134)**	1.03	1.01−1.06	**0.003**
**IL-10 (n=135)**	1.63	1.17−2.26	**0.003**
**IL-1ra (n=135)**	1.72	1.12−2.63	**0.012**
**IL-1β (n=135)**	2.49	1.32−4.70	**0.005**
**IL-6 (n=135)**	1.33	0.99−1.80	**0.055**
**IL-8 (n=135)**	1.75	1.24−2.47	**0.001**

Levels of biomarkers were log10 transformed before statistical analysis. Number of patients with available data are indicated in parenthesis. Abbreviations: CI, confidence interval; FiO2, fraction of inspired oxygen; PaO2, partial pressure of oxygen in arterial blood; SOFA, Sequential Organ Failure Assessment; qSOFA, quick Sequential Organ Failure Assessment. Statistically significant values are in bold character.

The c-statistics, sensitivity and specificity of each of these predictors are reported in **Table 7**. PTX3 showed the highest apparent c-statistic (0.716; 95% CI: 0.636−0.796) and sensitivity and specificity (threshold: 240 ng/ml; sensitivity: 48.4%, 95% CI: 31.9−64.9; specificity: 89.9%, 95% CI: 82.7−97.0) in our cohort of patients.

**Table 7:** Harrel C-statistics, sensitivity and specificity of each predictor included in the multivariable Cox model of 90-days mortality.

	*Threshold*	*C-statistic[95% CI]*	*Sensitivity (%)[95% CI]*	*Specificity (%)[95% CI]*
Age	87 years	0.60 [0.50−0.70]	29.0 [14.3−43.7]	91.3 [84.6−98.0]
**Blood urea nitrogen**	117 mg/dL	0.66 [0.56−0.76]	50.9 [34.4−67.4]	86.8 [78.7−94.8]
**PTX3**	240 ng/ml	0.72 [0.64−0.80]	48.4 [31.9−64.9]	89.9 [82.7−97.]
**IL-6**	175 pg/ml	0.62 [0.53−0.71]	63.3 [47.4−79.2]	65.2 [53.9−76.5]
**IL-18**	526 pg/ml	0.58 [0.48−0.67]	42.2 [25.9−58.5]	84.1 [75.4−92.7]

Reported thresholds were calculated for each predictor once dichotomized based on the maximally selected rank statistic. Abbreviations: CI, confidence interval.

Multivariable model specifications, including the baseline hazard at 90 days, are reported in **Table 8**. There was no evidence of violation of the proportionality of hazard assumption (*p*=0.192) and the Groennesby and Borgan test showed a reasonable model fit (*p*=0.783). The calibration plot showed good internal calibration (**Figure S1**), as indicated by a calibration slope equal to 1.146 (95% CI: 0.605−1.687) with an intercept not significantly different than zero (*p*=0.876). The Harrel c-statistic was 0.808 (95% CI: 0.734−0.882), indicating good discrimination performance.

**Table 8:** Multivariable Cox model of 90-days mortality: full model specification^1.^.

	β coefficient	95% CI	*p*-value
**Age**	+0.066	+0.029 to +0.103	**0.001**
**Blood urea nitrogen**	+0.008	+0.003 to +0.012	**<0.001**
**PTX3**	+1.343	+0.630 to +2.056	**<0.001**
**IL-6**	-4.961	-7.583 to -2.339	**<0.001**
**IL-18**	-3.437	-5.355 to -1.518	**<0.001**
IL-6*IL-18 (interaction term)	+1.903	+0.926 to +2.880	**<0.001**

^1^ H_0_ = 0.3894 (90-days baseline hazard). Values of PTX3, IL-6 and IL-18 were log10 transformed before analysis. Abbreviations: CI, confidence interval. Statistically significant values are in bold character.

We built a preliminary prognostic index by multiplying the multivariable model beta coefficients (including the interaction) by each patient’s characteristics:


$$Prognostic\;Index=0.066\times Age+1.343\times log_{10}(\text{PTX}3)-4.961\times \;log_{10}(\text{IL}-6)\;-3.437\times \;log_{10}(\text{IL}-18)+0.008\times Urea+1.903\times log_{10}(\text{IL}-18)\times log_{10}(\text{IL}-6)$$

**Figure 4:**
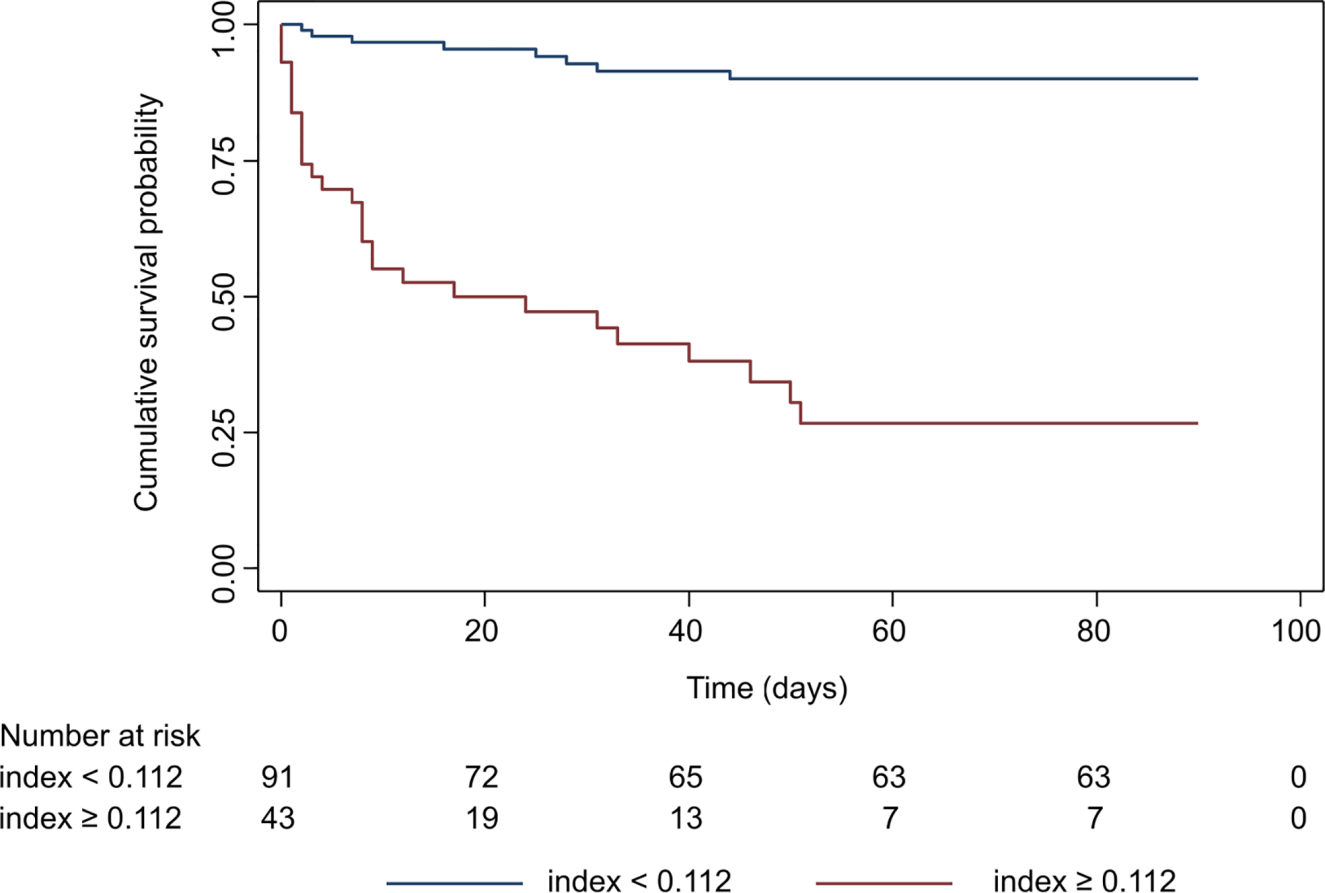
Kaplan-Meier survival curve of the prognostic index once dichotomized by maximally selected rank statistics. Patients were categorized in high and low mortality risk groups based on the threshold value of the prognostic index. Survival in the two groups was analyzed by Log-rank test (*p*<0.0001).

A nomogram plot associating each possible score with the individual 90-days risk of death is provided in **Figure S2**. The index was then dichotomized by applying the optimal threshold derived by maximally selected rank-statistics (threshold value = 0.112, corresponding to a predicted 90-days risk of death of 35.3%). This allowed patient stratification into high *vs.* low 90-days risk of death. The Kaplan Meier curves of patients belonging to the two groups are reported in **Figure 4**. Patients categorized by the index in the high mortality group had an observed cumulative 90-days risk of death of 73.3% (95% CI: 57.5−87.0), whereas patients in the low mortality group had a cumulative risk of 10.0% (95% CI: 5.12−19.1). The hazard ratio corresponding to the high mortality group was 12.0 (95% CI: 5.41−26.5). The sensitivity (76.9%; 95% CI: 62.8−91.0) and specificity (91.2%; 95% CI: 84.4−97.9) associated with this threshold showed a good performance of this classification rule in our sample of patients.

### Comparison of the prognostic index with SOFA and qSOFA

SOFA and qSOFA scores were used to identify patients with sepsis according with the Sepsis-3 criteria, however it is well known that this scoring system is also useful in predicting the clinical outcomes of critically ill patients (77, 78). We thus compared formally the AUC of our preliminary prognostic index with that of SOFA and qSOFA scores. The 90-days AUC of our preliminary index was 0.863 (95% CI: 0.780−0.945). The 90-days AUC of SOFA score was 0.727 (95% CI: 0.613−0.840), while the AUC for qSOFA at the same time-point was 0.660 (95% CI: 0.558−0.762). The AUC of our preliminary index was significantly greater than both SOFA (p=0.021) and qSOFA scores (*p*<0.001) in our cohort of patients, meaning that it was able to discriminate between patients dying from those surviving more accurately than the current gold standards.

## Discussion

Aim of this study was to evaluate the prognostic value of molecules of the innate immune response, namely PTX3 and sIL-1R2, in association with a set of pro- and anti-inflammatory cytokines (IL-1β, IL-6, IL-8, IL-10, IL-18, TNF-α and IL-1ra) and clinical parameters, in patients presenting at the Emergency Department with a suspicious or diagnosis of sepsis defined according to the Sepsis-3 criteria (65). At ED arrival, a progressive increase in the circulating levels of PTX3, sIL-1R2 and both pro- and anti-inflammatory cytokines was observed from non-sepsis to sepsis and septic shock patients. In univariable Cox analysis, PTX3, sIL-1R2 and all the cytokines analyzed in this study, except IL-18, were significant predictors of 90-days mortality. The combination of circulating levels of PTX3, IL-6 and IL-18, with age and blood urea nitrogen, constituted a preliminary prognostic index of 90-days mortality with high sensitivity and specificity, and more efficient than the gold standards SOFA and qSOFA scores, providing a useful tool to stratify sepsis patients on arrival at the ED.

The emergency department plays a pivotal role in the prompt recognition of septic status and activation of optimal therapeutic approaches (79–81). Biomarkers predicting mortality risk would represent invaluable tools to quickly provide patients with the most appropriate hospital care (82, 83). The biomarkers described here are in principle amenable to evolution to rapid point-of-care tests better addressing emergency department needs. Different platforms are available or under development, potentially useful in this context, including the ELLA microfluidic immunoassay system used in our work or multiplexed label-free biosensors (84–86)

Several reports addressed the role of PTX3 as a biomarker of sepsis (29, 31, 33–40), while scanty and contrasting data are available on sIL-1R2 (44–49), and a general consensus on its role in sepsis has not yet been reached. The present work has the merit to analyze patients arriving at the ED and diagnosed according to the latest Sepsis-3 definition, without excluding any pre-existing comorbidity, including cancer. The model developed can be used for the early prediction of 90-days mortality risk, a timing no longer affected by the different therapies. PTX3 and sIL-1R2 circulating levels were increased, reflecting the augmented tissue damage and inflammation associated with the increased severity. In addition, both molecules strongly correlated with SOFA score in sepsis and septic shock patients (PTX3: r=0.44, *p*<0.0001; sIL-1R2: r=0.35, *p*<0.0001), confirming data obtained in earlier studies (31, 44), as well as with PCT, CRP and clinical parameters of severity. These data demonstrated that PTX3 and sIL-1R2 could be useful in monitoring the severity of disease in early hours of admission to ED.

The role of the immune system in sepsis is generally recognized and involves multiple processes, ranging from activation of pro-inflammatory mechanisms to immunosuppression, often occurring simultaneously (87–89). Early activation of the inflammatory response is involved in the pathogenesis of sepsis, and the altered levels of several cytokines observed in septic patients compared to healthy controls are likely involved in the organ injury observed in sepsis (9, 90). In our cohort of patients, we observed significant changes in the levels of the panel of pro-inflammatory (IL-1β, IL-6, IL-8, IL-18, TNF-α) and anti-inflammatory (IL-10, IL-1ra) cytokines. All the molecules were augmented in patients compared to HC and correlated with SOFA score, evidencing, as for PTX3 and sIL-1R2, the progressive increase of their circulating levels with the severity of disease. The concomitant upregulation of PTX3, CRP and pro-inflammatory cytokines (IL-6, IL-8, TNF-α) on one side, and of the anti-inflammatory molecules sIL-1R2, IL-10 and IL-1ra on the other side, further supports the coexistence of pro-inflammatory and immunosuppressive mechanisms throughout the host response to sepsis.

After 5 days of hospitalization, a general decrease of PTX3 circulating levels was observed, in parallel with an improvement of patients’ conditions and a reduction of the inflammation and/or infection burden. In parallel, a reduction of CRP, PCT and cytokines levels was observed in the cohort of patients, together with the general improvement of clinical parameters. On the contrary, no significant changes were recorded in circulating levels of sIL-1R2 in this time frame. Considering that sIL-1R2 is a key negative regulator of the IL-1 system, the maintenance of high levels of the molecule could tightly regulate the responses to IL-1 family members, contributing to protect from an exaggerated inflammatory response (26).

PTX3, sIL-1R2 and all cytokines investigated in our study were significantly higher in non-survivors compared to 90-days survivors, and were strong predictors of mortality. These results are in agreement with several studies reporting the association of cytokines such as IL-6 or IL-18 with poor outcome in septic patients (91, 92). In another study of a small cohort of sepsis, severe sepsis and shock patient admitted to ICU, the levels of IL-6, IL-8 and IL-18 resulted higher in non-survivors compared to survivors, although following multivariable logistic regression analysis only IL-18 remained related to mortality (93). In addition, a positive synergistic effect has been observed between IL-18 and IL-6 that likely contributes to the association of these molecules with mortality, despite IL-18 not being a significant predictor of mortality by Cox proportional analysis. Our study was limited to a selected number of soluble mediators, and we are aware that other molecules could also play relevant roles. Growing interest has been observed for circulating antagonists of the IL-1 pathway, such as IL-18 binding protein (IL-18BP) and IL-1R4, also known as ST2 (94, 95), even for possible therapeutic implications (96). In a sub-group of patients, we measured both IL-18BP and IL-1R4: the two molecules were mildly increased in patients compared to healthy controls (data not shown). Although an increasing trend was seen from non-sepsis to sepsis and shock patients, IL-18BP and IL-1R4 were not significantly associated with mortality, in contrast to what reported by others (93, 96–98). A future comprehensive analysis of all the different molecules belonging to the IL-1 pathway would be helpful to fully define the strength of this essential family of inflammatory mediators in assessing sepsis patients.

Overall, the literature shows that none of the cytokines is a robust biomarker of mortality by itself (6, 11, 99). For this reason, the most recent investigations have mainly focused on the analysis of a combination of multiple biomarkers to predict outcome in sepsis patients (12–18). In a recent prospective observational analysis on septic patients arriving at the ED, Song et al. showed that the combination of PTX3, IL-6, PCT and lactate was effective in predicting 28-days mortality with a good performance and better than SOFA score (100, 101). IL-6 alone, in combination with other molecules (102–104) or with neutrophil-to-lymphocyte ratio can be a marker of mortality in sepsis patients (74). Similarly, Matsumoto et al. suggested the involvement of a cytokine network including IL-6, IL-8, MCP-1 and IL-10 in the acute phase of sepsis and proposed the development of a combined score including IL-6 which is significantly correlated with prognosis (90). On the same line, our data argue in favor of the usefulness of testing a combination of multiple biomarkers to improve the prognostic capacity.

In multivariable Cox regression analysis, the combination of PTX3, urea, IL-6 and IL-18 levels with age showed the best prognostic value. Since this study includes a relatively low number of patients and death events, we ensured to select a parsimonious model with a very conservative threshold for significance (*p*<0.005). This model showed good internal calibration properties and could predict 90-days death significantly better than the SOFA score in our sample of patients, though these findings should be externally validated in an independent cohort in order to be generalizable.

Despite these limitations, these results could help to generate new hypotheses on the prognostic, but also etiological, role of IL-6 and IL-18 in sepsis. Similar to the study by Mierzchala-Pasierb et al. (105), we did not observe significant differences of IL-18 levels in survivors *vs.* non-survivors. However, both IL-18 and IL-6 were significant predictors of mortality in the full model. Additionally, when PTX3, urea levels and age were left unchanged, there was a highly significant interaction (positive synergistic effect) between IL-6 and IL-18, meaning there was a non-linear association between these two variables and 90-days mortality. This suggests that, although at low levels, IL-6 and IL-18 do not significantly affect the risk of mortality, thus, after a certain threshold, each increase becomes very importantly associated with the predicted mortality. This could perhaps be explained by the fact that IL-18 can induce the production of huge quantities of IL-6 from a variety of cell types through the activation of the NLRP3 inflammasome and caspase 1, effectively amplifying other pro-inflammatory signals (106). In our study, the variance of IL-18 values in sepsis patients was much lower (range from 6 pg/ml to about 2000 pg/ml) than what was observed for IL-6 (from 6 pg/ml to almost 1 million pg/ml). This may point toward a different magnitude of production of IL-6 in certain high-risk septic patients, in presence of relatively similar levels of IL-18. Further studies are necessary to characterize the physio-pathological role of these cytokines in sepsis patients.

Besides the aforementioned low number of participants, its monocentric nature, and the need for external validation, this study has other limitations. Patient demographics in our study cohort differed from other sepsis studies, including enrollment of patients at ED admission instead of later in the ICU setting. This adds novelty to our study, but also adds heterogeneity to our results. Some clinical data are missing for a fraction of patients, such as lactate and PCT levels, and we did not have other clinical scores, such as Simplified Acute Physiology Score (SAPS) II, SAPS III, and/or Acute Physiology and Chronic Health Evaluation II (APACHE II) scores. In our cohort, 31% of sepsis patients and 39% of septic shock patients had a malignancy as comorbidity, whereas many studies exclude malignancy-linked sepsis. However, we believe that considering all sepsis cases regardless of comorbidities could have added diagnostic value in the ED setting. Given the different comorbidities in our cohort of patients, the inflammatory markers such as CRP and PCT may be also influenced, potentially affecting the results; however, the reduction observed in patients after 5 days of hospitalization suggested that comorbidities had a limited impact on the overall inflammatory status of the patients. These preliminary results need to be confirmed with multicentric studies involving larger cohorts of patients. Overall, the increasing development of point-of-care testing systems for the rapid and accurate measurement of circulating molecules makes the evaluation of multiple soluble mediators feasible even in the emergency room (85, 86).

In conclusion, we concur with other reports that a combination of inflammatory mediators and clinical parameters can improve risk stratification of sepsis patients. Our data indicate that high levels of PTX3 in plasma of patients with suspected sepsis admitted to the emergency room can be used as a prognostic marker for risk stratification. In addition, measurement of PTX3 in combination with cytokine levels (IL-6 and IL-18), and other routine clinical parameters rapidly available in ED (e.g., age, urea), can provide a promising approach for 90-days mortality prediction for sepsis patients, better than the use of a single biomarker.

## Data availability statement

The raw data supporting the conclusions of this article will be made available by the authors, without undue reservation.

## Ethics statement

The studies involving human participants were reviewed and approved by the Institutional Review Board of Humanitas Hospital (Approval n° 820/18). The patients/participants provided their written informed consent to participate in this study.

## Author contributions

SD, AV, AlbM, and BB made substantial contributions to the conception and design of the study; AD, AleM, DPv, CF, and DC collected patient samples and clinical data; SD, RL, MSi, SV and RS-G performed the experiments; SD, DP, SM, MSt and SB performed the data analyses; SD, DP, AD, SB, AV, AlbM, MSt and BB interpreted the results; SD, DP, AlbM, and BB wrote the paper. All authors were involved in the analysis and interpretation of data, drafting the manuscript and revising it critically, and approved the final version.

## Funding

SD was supported by European Sepsis Academy/Innovative Training Networks (ESA/ITN) from the European Commission (H2020-MSCA-ITN-2015, Grant agreement Number: 676129). RS-G acknowledge financial support from Fundação para a Ciência e a Tecnologia (FCT) for Ph.D. grant PD/BD/114138/2016. This study has received funding from Associazione Italiana Ricerca sul Cancro (AIRC) [Special Program Metastatic disease: the key unmet need in oncology AIRC 5X1000 grant n° 21147 and “Regulatory pathways of myeloid cells, inflammation and cancer” grant IG-2019 N. 23465].

## Acknowledgments

We thank all the physicians, nurses, and laboratory personnel of the Emergency Department and wards of Humanitas Research Hospital for their active collaboration. Also, a special thanks to all the patients who participated in this study.

## Conflict of interest

AlbM and BB are inventors of a patent (EP20182181) on PTX3 and obtain royalties on related reagents.

The remaining authors declare that the research was conducted in the absence of any commercial or financial relationships that could be construed as a potential conflict of interest.

## Publisher’s note

All claims expressed in this article are solely those of the authors and do not necessarily represent those of their affiliated organizations, or those of the publisher, the editors and the reviewers. Any product that may be evaluated in this article, or claim that may be made by its manufacturer, is not guaranteed or endorsed by the publisher.

## References

[1] Saeed K Wilson DC Bloos F Schuetz P van der Does Y Melander O. The early identification of disease progression in patients with suspected infection presenting to the emergency department: A multi-centre derivation and validation study. Crit Care (2019) 23(1):40. doi: 10.1186/s13054-019-2329-5 30736862 PMC6368690

[2] Kumar S Tripathy S Jyoti A Singh SG. Recent advances in biosensors for diagnosis and detection of sepsis: A comprehensive review. Biosens Bioelectron (2019) 124-125:205–15. doi: 10.1016/j.bios.2018.10.034 30388563

[3] Rudd KE Johnson SC Agesa KM Shackelford KA Tsoi D Kievlan DR. Global, regional, and national sepsis incidence and mortality, 1990-2017: Analysis for the global burden of disease study. Lancet (2020) 395(10219):200–11. doi: 10.1016/S0140-6736(19)32989-7 PMC6970225 31954465

[4] Cecconi M Evans L Levy M Rhodes A. Sepsis and septic shock. Lancet (2018) 392(10141):75–87. doi: 10.1016/S0140-6736(18)30696-2 29937192

[5] Ward NS Levy MM. Sepsis: Definitions, pathophysiology and the challenge of bedside management. Humana Press (2019). doi: 10.1007/978-3-319-48470-9

[6] Pierrakos C Vincent JL. Sepsis biomarkers: A review. Crit Care (2010) 14(1):R15. doi: 10.1186/cc8872 20144219 PMC2875530

[7] Kyriazopoulou E Giamarellos-Bourboulis EJ. Antimicrobial stewardship using biomarkers: Accumulating evidence for the critically ill. Antibiot (Basel) (2022) 11(3):367. doi: 10.3390/antibiotics11030367 PMC8944654 35326830

[8] van Engelen TSR Wiersinga WJ Scicluna BP van der Poll T. Biomarkers in sepsis. Crit Care Clin (2018) 34(1):139–52. doi: 10.1016/j.ccc.2017.08.010 29149935

[9] D’Onofrio V Heylen D Pusparum M Grondman I Vanwalleghem J Meersman A. A prospective observational cohort study to identify inflammatory biomarkers for the diagnosis and prognosis of patients with sepsis. J Intensive Care (2022) 10(1):13. doi: 10.1186/s40560-022-00602-x 35264246 PMC8905560

[10] Niederman MS Baron RM Bouadma L Calandra T Daneman N DeWaele J. Initial antimicrobial management of sepsis. Crit Care (2021) 25(1):307. doi: 10.1186/s13054-021-03736-w 34446092 PMC8390082

[11] Pierrakos C Velissaris D Bisdorff M Marshall JC Vincent JL. Biomarkers of sepsis: Time for a reappraisal. Crit Care (2020) 24(1):287. doi: 10.1186/s13054-020-02993-5 32503670 PMC7273821

[12] Mearelli F Fiotti N Giansante C Casarsa C Orso D De Helmersen M. Derivation and validation of a biomarker-based clinical algorithm to rule out sepsis from noninfectious systemic inflammatory response syndrome at emergency department admission: A multicenter prospective study. Crit Care Med (2018) 46(9):1421–9. doi: 10.1097/CCM.0000000000003206 29742588

[13] Teggert A Datta H Ali Z. Biomarkers for point-of-Care diagnosis of sepsis. Micromachines (Basel) (2020) 11(3):286. doi: 10.3390/mi11030286 PMC7143187 32164268

[14] Kyriazopoulou E Poulakou G Giamarellos-Bourboulis EJ. Biomarkers in sepsis: Can they help improve patient outcome? Curr Opin Infect Dis (2021) 34(2):126–34. doi: 10.1097/qco.0000000000000707 33534419

[15] Gao L Shi Q Li H Guo Q Yan J Zhou L. Prognostic value of the combined variability of mean platelet volume and neutrophil percentage for short-term clinical outcomes of sepsis patients. Postgrad Med (2021) 133(6):604–12. doi: 10.1080/00325481.2020.1823137 32912023

[16] Kim H Hur M Moon HW Yun YM Di Somma S Network G. Multi-marker approach using procalcitonin, presepsin, galectin-3, and soluble suppression of tumorigenicity 2 for the prediction of mortality in sepsis. Ann Intensive Care (2017) 7(1):27. doi: 10.1186/s13613-017-0252-y 28271449 PMC5340789

[17] Shukeri W Ralib AM Abdulah NZ Mat-Nor MB. Sepsis mortality score for the prediction of mortality in septic patients. J Crit Care (2018) 43:163–8. doi: 10.1016/j.jcrc.2017.09.009 28903084

[18] Walborn A Rondina M Fareed J Hoppensteadt D. Development of an algorithm to predict mortality in patients with sepsis and coagulopathy. Clin Appl Thromb Hemost (2020) 26:1076029620902849. doi: 10.1177/1076029620902849 32129085 PMC7288806

[19] Garlanda C Bottazzi B Bastone A Mantovani A. Pentraxins at the crossroads between innate immunity, inflammation, matrix deposition, and female fertility. Annu Rev Immunol (2005) 23:337–66. doi: 10.1146/annurev.immunol.23.021704.115756 15771574

[20] Garlanda C Bottazzi B Magrini E Inforzato A Mantovani A. Ptx3, a humoral pattern recognition molecule, in innate immunity, tissue repair, and cancer. Physiol Rev (2018) 98(2):623–39. doi: 10.1152/physrev.00016.2017 PMC5985957 29412047

[21] Bottazzi B Doni A Garlanda C Mantovani A. An integrated view of humoral innate immunity: Pentraxins as a paradigm. Annu Rev Immunol (2010) 28:157–83. doi: 10.1146/annurev-immunol-030409-101305 19968561

[22] Jeannin P Bottazzi B Sironi M Doni A Rusnati M Presta M. Complexity and complementarity of outer membrane protein a recognition by cellular and humoral innate immunity receptors. Immunity (2005) 22(5):551–60. doi: 10.1016/j.immuni.2005.03.008 15894273

[23] Doni A Musso T Morone D Bastone A Zambelli V Sironi M. An acidic microenvironment sets the humoral pattern recognition molecule Ptx3 in a tissue repair mode. J Exp Med (2015) 212(6):905–25. doi: 10.1084/jem.20141268 PMC4451130 25964372

[24] Garlanda C Riva F Bonavita E Gentile S Mantovani A. Decoys and regulatory “Receptors” of the il-1/Toll-Like receptor superfamily. Front Immunol (2013) 4:180. doi: 10.3389/fimmu.2013.00180 23847621 PMC3705552

[25] Garlanda C Riva F Bonavita E Mantovani A. Negative regulatory receptors of the il-1 family. Semin Immunol (2013) 25(6):408–15. doi: 10.1016/j.smim.2013.10.019 24239046

[26] Molgora M Supino D Mantovani A Garlanda C. Tuning inflammation and immunity by the negative regulators il-1r2 and il-1r8. Immunol Rev (2018) 281(1):233–47. doi: 10.1111/imr.12609 PMC5922415 29247989

[27] Peters VA Joesting JJ Freund GG. Il-1 receptor 2 (Il-1r2) and its role in immune regulation. Brain Behav Immun (2013) 32:1–8. doi: 10.1016/j.bbi.2012.11.006 23195532 PMC3610842

[28] Slack JL Schooley K Bonnert TP Mitcham JL Qwarnstrom EE Sims JE. Identification of two major sites in the type I interleukin-1 receptor cytoplasmic region responsible for coupling to pro-inflammatory signaling pathways. J Biol Chem (2000) 275(7):4670–8. doi: 10.1074/jbc.275.7.4670 10671496

[29] Muller B Peri G Doni A Torri V Landmann R Bottazzi B. Circulating levels of the long pentraxin Ptx3 correlate with severity of infection in critically ill patients. Crit Care Med (2001) 29(7):1404–7. doi: 10.1097/00003246-200107000-00017 11445697

[30] Azzurri A Sow OY Amedei A Bah B Diallo S Peri G. Ifn-Gamma-Inducible protein 10 and pentraxin 3 plasma levels are tools for monitoring inflammation and disease activity in mycobacterium tuberculosis infection. Microbes Infect (2005) 7(1):1–8. doi: 10.1016/j.micinf.2004.09.004 15716076

[31] Caironi P Masson S Mauri T Bottazzi B Leone R Magnoli M. Pentraxin 3 in patients with severe sepsis or shock: The albios trial. Eur J Clin Invest (2017) 47(1):73–83. doi: 10.1111/eci.12704 27864924 PMC5414835

[32] Mairuhu AT Peri G Setiati TE Hack CE Koraka P Soemantri A. Elevated plasma levels of the long pentraxin, pentraxin 3, in severe dengue virus infections. J Med Virol (2005) 76(4):547–52. doi: 10.1002/jmv.20397 15977234

[33] Porte R Davoudian S Asgari F Parente R Mantovani A Garlanda C. The long pentraxin Ptx3 as a humoral innate immunity functional player and biomarker of infections and sepsis. Front Immunol (2019) 10:794. doi: 10.3389/fimmu.2019.00794 31031772 PMC6473065

[34] Hamed S Behnes M Pauly D Lepiorz D Barre M Becher T. Diagnostic value of pentraxin-3 in patients with sepsis and septic shock in accordance with latest sepsis-3 definitions. BMC Infect Dis (2017) 17(1):554. doi: 10.1186/s12879-017-2606-3 28793880 PMC5550951

[35] Jie H Li Y Pu X Ye J. Pentraxin 3, a predicator for 28-day mortality in patients with septic shock. Am J Med Sci (2017) 353(3):242–6. doi: 10.1016/j.amjms.2017.01.003 28262210

[36] Lee YT Gong M Chau A Wong WT Bazoukis G Wong SH. Pentraxin-3 as a marker of sepsis severity and predictor of mortality outcomes: A systematic review and meta-analysis. J Infect (2018) 76(1):1–10. doi: 10.1016/j.jinf.2017.10.016 29174966

[37] Mauri T Bellani G Patroniti N Coppadoro A Peri G Cuccovillo I. Persisting high levels of plasma pentraxin 3 over the first days after severe sepsis and septic shock onset are associated with mortality. Intensive Care Med (2010) 36(4):621–9. doi: 10.1007/s00134-010-1752-5 20119647

[38] Chen H Li T Yan S Liu M Liu K Zhang H. Pentraxin-3 is a strong biomarker of sepsis severity identification and predictor of 90-day mortality in intensive care units *Via* sepsis 3.0 definitions. Diagn (Basel) (2021) 11(10):1906. doi: 10.3390/diagnostics11101906 PMC8534382 34679604

[39] Wang G Jiang C Fang J Li Z Cai H. Pentraxin-3 as a predictive marker of mortality in sepsis: An updated systematic review and meta-analysis. Crit Care (2022) 26(1):167. doi: 10.1186/s13054-022-04032-x 35676730 PMC9175505

[40] Uusitalo-Seppala R Huttunen R Aittoniemi J Koskinen P Leino A Vahlberg T. Pentraxin 3 (Ptx3) is associated with severe sepsis and fatal disease in emergency room patients with suspected infection: A prospective cohort study. PloS One (2013) 8(1):e53661. doi: 10.1371/journal.pone.0053661 23341967 PMC3544919

[41] Arend WP Malyak M Smith MF Jr. Whisenand TD Slack JL Sims JE. Binding of il-1 alpha, il-1 beta, and il-1 receptor antagonist by soluble il-1 receptors and levels of soluble il-1 receptors in synovial fluids. J Immunol (1994) 153(10):4766–74. 7963543

[42] Dinarello CA. Biologic basis for interleukin-1 in disease. Blood (1996) 87(6):2095–147. doi: 10.1182/blood.V87.6.2095.bloodjournal8762095 8630372

[43] Jouvenne P Vannier E Dinarello CA Miossec P. Elevated levels of soluble interleukin-1 receptor type ii and interleukin-1 receptor antagonist in patients with chronic arthritis: Correlations with markers of inflammation and joint destruction. Arthritis Rheum (1998) 41(6):1083–9. doi: 10.1002/1529-0131(199806)41:6<1083::AID-ART15>3.0.CO;2-9 9627018

[44] Muller B Peri G Doni A Perruchoud AP Landmann R Pasqualini F. High circulating levels of the il-1 type ii decoy receptor in critically ill patients with sepsis: Association of high decoy receptor levels with glucocorticoid administration. J Leukoc Biol (2002) 72(4):643–9. doi: 10.1189/jlb.72.4.643 12377932

[45] van der Poll T de Waal Malefyt R Coyle SM Lowry SF. Antiinflammatory cytokine responses during clinical sepsis and experimental endotoxemia: Sequential measurements of plasma soluble interleukin (Il)-1 receptor type ii, il-10, and il-13. J Infect Dis (1997) 175(1):118–22. doi: 10.1093/infdis/175.1.118 8985204

[46] Giri JG Wells J Dower SK McCall CE Guzman RN Slack J. Elevated levels of shed type ii il-1 receptor in sepsis. potential role for type ii receptor in regulation of il-1 responses. J Immunol (1994) 153(12):5802–9. 7989776

[47] Pruitt JH Welborn MB Edwards PD Harward TR Seeger JW Martin TD. Increased soluble interleukin-1 type ii receptor concentrations in postoperative patients and in patients with sepsis syndrome. Blood (1996) 87(8):3282–8. doi: 10.1182/blood.V87.8.3282.bloodjournal8783282 8605344

[48] van Deuren M van der Ven-Jongekrijg J Vannier E van Dalen R Pesman G Bartelink AK. The pattern of interleukin-1beta (Il-1beta) and its modulating agents il-1 receptor antagonist and il-1 soluble receptor type ii in acute meningococcal infections. Blood (1997) 90(3):1101–8. doi: 10.1182/blood.V90.3.1101 9242541

[49] Pruitt JH Copeland EM Moldawer LL 3rd. Interleukin-1 and interleukin-1 antagonism in sepsis, systemic inflammatory response syndrome, and septic shock. Shock (1995) 3(4):235–51. doi: 10.1097/00024382-199504000-00001 7600191

[50] Hotchkiss RS Karl IE. The pathophysiology and treatment of sepsis. N Engl J Med (2003) 348(2):138–50. doi: 10.1056/NEJMra021333 12519925

[51] Seeley EJ Bernard GR. Therapeutic targets in sepsis: Past, present, and future. Clin Chest Med (2016) 37(2):181–9. doi: 10.1016/j.ccm.2016.01.015 27229636

[52] van der Poll T Opal SM. Host-pathogen interactions in sepsis. Lancet Infect Dis (2008) 8(1):32–43. doi: 10.1016/S1473-3099(07)70265-7 18063412

[53] Chousterman BG Swirski FK Weber GF. Cytokine storm and sepsis disease pathogenesis. Semin Immunopathol (2017) 39(5):517–28. doi: 10.1007/s00281-017-0639-8 28555385

[54] Grobmyer SR Lin E Lowry SF Rivadeneira DE Potter S Barie PS. Elevation of il-18 in human sepsis. J Clin Immunol (2000) 20(3):212–5. doi: 10.1023/a:1006641630904 10941829

[55] Friedman G Jankowski S Marchant A Goldman M Kahn RJ Vincent JL. Blood interleukin 10 levels parallel the severity of septic shock. J Crit Care (1997) 12(4):183–7. doi: 10.1016/s0883-9441(97)90030-7 9459114

[56] Gardlund B Sjolin J Nilsson A Roll M Wickerts CJ Wretlind B. Plasma levels of cytokines in primary septic shock in humans: Correlation with disease severity. J Infect Dis (1995) 172(1):296–301. doi: 10.1093/infdis/172.1.296 7797935

[57] Gogos CA Drosou E Bassaris HP Skoutelis A. Pro- versus anti-inflammatory cytokine profile in patients with severe sepsis: A marker for prognosis and future therapeutic options. J Infect Dis (2000) 181(1):176–80. doi: 10.1086/315214 10608764

[58] van Dissel JT van Langevelde P Westendorp RG Kwappenberg K Frolich M. Anti-inflammatory cytokine profile and mortality in febrile patients. Lancet (1998) 351(9107):950–3. doi: 10.1016/S0140-6736(05)60606-X 9734942

[59] Ertel W Kremer JP Kenney J Steckholzer U Jarrar D Trentz O. Downregulation of proinflammatory cytokine release in whole blood from septic patients. Blood (1995) 85(5):1341–7. doi: 10.3171/jns.1990.72.4.0572 7858264

[60] Rigato O Salomao R. Impaired production of interferon-gamma and tumor necrosis factor-alpha but not of interleukin 10 in whole blood of patients with sepsis. Shock (2003) 19(2):113–6. doi: 10.1097/00024382-200302000-00004 12578117

[61] Sinistro A Almerighi C Ciaprini C Natoli S Sussarello E Di Fino S. Downregulation of Cd40 ligand response in monocytes from sepsis patients. Clin Vaccine Immunol (2008) 15(12):1851–8. doi: 10.1128/CVI.00184-08 PMC2593171 18945879

[62] Weighardt H Heidecke CD Emmanuilidis K Maier S Bartels H Siewert JR. Sepsis after major visceral surgery is associated with sustained and interferon-Gamma-Resistant defects of monocyte cytokine production. Surgery (2000) 127(3):309–15. doi: 10.1067/msy.2000.104118 10715987

[63] Oberholzer A Souza SM Tschoeke SK Oberholzer C Abouhamze A Pribble JP. Plasma cytokine measurements augment prognostic scores as indicators of outcome in patients with severe sepsis. Shock (2005) 23(6):488–93. doi: 10.1097/01.shk.0000163802.46355.59 15897799

[64] Pinsky MR Vincent JL Deviere J Alegre M Kahn RJ Dupont E. Serum cytokine levels in human septic shock. relation to multiple-system organ failure and mortality. Chest (1993) 103(2):565–75. doi: 10.1378/chest.103.2.565 8432155

[65] Singer M Deutschman CS Seymour CW Shankar-Hari M Annane D Bauer M. The third international consensus definitions for sepsis and septic shock (Sepsis-3). JAMA (2016) 315(8):801–10. doi: 10.1001/jama.2016.0287 PMC4968574 26903338

[66] Knoflach M Kiechl S Mantovani A Cuccovillo I Bottazzi B Xu Q. Pentraxin-3 as a marker of advanced atherosclerosis results from the bruneck, army and arfy studies. PloS One (2012) 7(2):e31474. doi: 10.1371/journal.pone.0031474 22319633 PMC3272046

[67] Royston P Sauerbrei W. Multivariable model - building: A pragmatic approach to regression anaylsis based on fractional polynomials for modelling continuous variables. Chichester, England: John Wiley & Sons (2008).

[68] Moons KG Altman DG Reitsma JB Collins GS. New guideline for the reporting of studies developing, validating, or updating a prediction model. Clin Chem (2015) 61(3):565–6. doi: 10.1373/clinchem.2014.237883 25874290

[69] Gronnesby JK Borgan O. A method for checking regression models in survival analysis based on the risk score. Lifetime Data Anal (1996) 2(4):315–28. doi: 10.1007/BF00127305 9384628

[70] Hothorn T Lausen B. On the exact distribution of maximally selected rank statistics. Comput Stat Data Anal (2003) 43(2):121–37. doi: 10.1016/S0167-9473(02)00225-6

[71] Kamarudin AN Cox T Kolamunnage-Dona R. Time-dependent roc curve analysis in medical research: Current methods and applications. BMC Med Res Methodol (2017) 17(1):53. doi: 10.1186/s12874-017-0332-6 28388943 PMC5384160

[72] Henriquez-Camacho C Losa J. Biomarkers for sepsis. BioMed Res Int (2014) 2014:547818. doi: 10.1155/2014/547818 24800240 PMC3985161

[73] So-Ngern A Leelasupasri S Chulavatnatol S Pummangura C Bunupuradah P Montakantikul P. Prognostic value of serum procalcitonin level for the diagnosis of bacterial infections in critically-ill patients. Infect Chemother (2019) 51(3):263–73. doi: 10.3947/ic.2019.51.3.263 PMC6779575 31583860

[74] Liu B Chen YX Yin Q Zhao YZ Li CS. Diagnostic value and prognostic evaluation of presepsin for sepsis in an emergency department. Crit Care (2013) 17(5):R244. doi: 10.1186/cc13070 24138799 PMC4056322

[75] Niederman MS. Biological markers to determine eligibility in trials for community-acquired pneumonia: A focus on procalcitonin. Clin Infect Dis (2008) 47(Supplement_3):S127–S32. doi: 10.1086/591393 18986278

[76] Petrovic J Turnic TN Zivkovic V Andjic M Draginic N Stojanovic A. Correlation of redox status with procalcitonin and c-reactive protein in septic patients. Oxid Med Cell Longev (2020) 2020:5147364. doi: 10.1155/2020/5147364 32952850 PMC7487118

[77] Gruyters I De Ridder T Bruckers L Geebelen L Gharmaoui S Callebaut I. Predictive value of serial evaluation of the sequential organ failure assessment (Sofa) score for intensive care unit mortality in critically ill patients with covid-19: A retrospective cohort study. Anaesthesiol Intensive Ther (2022) 54(1):3–11. doi: 10.5114/ait.2022.114048 35266376 PMC10156486

[78] Vincent JL de Mendonça A Cantraine F Moreno R Takala J Suter PM. Use of the sofa score to assess the incidence of organ Dysfunction/Failure in intensive care units: Results of a multicenter, prospective study. working group on “Sepsis-related problems” of the European society of intensive care medicine. Crit Care Med (1998) 26(11):1793–800. doi: 10.1097/00003246-199811000-00016 9824069

[79] Dellinger RP Levy MM Rhodes A Annane D Gerlach H Opal SM. Surviving sepsis campaign: International guidelines for management of severe sepsis and septic shock, 2012. Intensive Care Med (2013) 39(2):165–228. doi: 10.1007/s00134-012-2769-8 23361625 PMC7095153

[80] Mouncey PR Osborn TM Power GS Harrison DA Sadique MZ Grieve RD. Trial of early, goal-directed resuscitation for septic shock. N Engl J Med (2015) 372(14):1301–11. doi: 10.1056/NEJMoa1500896 25776532

[81] Levy MM Evans LE Rhodes A. The surviving sepsis campaign bundle: 2018 update. Crit Care Med (2018) 46(6):997–1000. doi: 10.1097/CCM.0000000000003119 29767636

[82] Simpson SQ. Sepsis biomarkers and physician judgment in the emergency room. Crit Care Med (2019) 47(11):1656–7. doi: 10.1097/CCM.0000000000003983 31609261

[83] Bloos F Ruddel H Thomas-Ruddel D Schwarzkopf D Pausch C Harbarth S. Effect of a multifaceted educational intervention for anti-infectious measures on sepsis mortality: A cluster randomized trial. Intensive Care Med (2017) 43(11):1602–12. doi: 10.1007/s00134-017-4782-4 28466151

[84] Albert Vega C Mommert M Boccard M Rimmele T Venet F Pachot A. Source of circulating pentraxin 3 in septic shock patients. Front Immunol (2018) 9:3048. doi: 10.3389/fimmu.2018.03048 30687307 PMC6338061

[85] Bonavia AS Samuelsen A Chroneos ZC Halstead ES. Comparison of rapid cytokine immunoassays for functional immune phenotyping. Front Immunol (2022) 13:940030. doi: 10.3389/fimmu.2022.940030 35860253 PMC9289684

[86] Bottazzi B Fornasari L Frangolho A Giudicatti S Mantovani A Marabelli F. Multiplexed label-free optical biosensor for medical diagnostics. J BioMed Opt (2014) 19(1):17006. doi: 10.1117/1.JBO.19.1.017006 24474511

[87] Hotchkiss RS Monneret G Payen D. Sepsis-induced immunosuppression: From cellular dysfunctions to immunotherapy. Nat Rev Immunol (2013) 13(12):862–74. doi: 10.1038/nri3552 PMC4077177 24232462

[88] Xiao H Siddiqui J Remick DG. Mechanisms of mortality in early and late sepsis. Infect Immun (2006) 74(9):5227–35. doi: 10.1128/IAI.01220-05 PMC1594860 16926416

[89] Rubio I Osuchowski MF Shankar-Hari M Skirecki T Winkler MS Lachmann G. Current gaps in sepsis immunology: New opportunities for translational research. Lancet Infect Dis (2019) 19(12):e422–e36. doi: 10.1016/s1473-3099(19)30567-5 31630991

[90] Matsumoto H Ogura H Shimizu K Ikeda M Hirose T Matsuura H. The clinical importance of a cytokine network in the acute phase of sepsis. Sci Rep (2018) 8(1):13995. doi: 10.1038/s41598-018-32275-8 30228372 PMC6143513

[91] Emmanuilidis K Weighardt H Matevossian E Heidecke CD Ulm K Bartels H. Differential regulation of systemic il-18 and il-12 release during postoperative sepsis: High serum il-18 as an early predictive indicator of lethal outcome. Shock (2002) 18(4):301–5. doi: 10.1097/00024382-200210000-00002 12392271

[92] Tschoeke SK Oberholzer A Moldawer LL. Interleukin-18: A novel prognostic cytokine in bacteria-induced sepsis. Crit Care Med (2006) 34(4):1225–33. doi: 10.1097/01.CCM.0000208356.05575.16 16540967

[93] Eidt MV Nunes FB Pedrazza L Caeran G Pellegrin G Melo DA. Biochemical and inflammatory aspects in patients with severe sepsis and septic shock: The predictive role of il-18 in mortality. Clin Chim Acta (2016) 453:100–6. doi: 10.1016/j.cca.2015.12.009 26683353

[94] De la Fuente M MacDonald TT Hermoso MA. The il-33/St2 axis: Role in health and disease. Cytokine Growth Factor Rev (2015) 26(6):615–23. doi: 10.1016/j.cytogfr.2015.07.017 26271893

[95] Dinarello CA Novick D Kim S Kaplanski G. Interleukin-18 and il-18 binding protein. Front Immunol (2013) 4:289. doi: 10.3389/fimmu.2013.00289 24115947 PMC3792554

[96] Novick D Schwartsburd B Pinkus R Suissa D Belzer I Sthoeger Z. A novel il-18bp Elisa shows elevated serum il-18bp in sepsis and extensive decrease of free il-18. Cytokine (2001) 14(6):334–42. doi: 10.1006/cyto.2001.0914 11497494

[97] Krychtiuk KA Stojkovic S Lenz M Brekalo M Huber K Wojta J. Predictive value of low interleukin-33 in critically ill patients. Cytokine (2018) 103:109–13. doi: 10.1016/j.cyto.2017.09.017 28974430

[98] Kyriakoudi A Rovina N Koltsida O Kostakou E Konstantelou E Kardara M. Weaning failure in critically ill patients is related to the persistence of sepsis inflammation. Diagnostics (2022) 12:92. doi: 10.3390/diagnostics12010092 PMC8774440 35054259

[99] Faix JD. Biomarkers of sepsis. Crit Rev Clin Lab Sci (2013) 50(1):23–36. doi: 10.3109/10408363.2013.764490 23480440 PMC3613962

[100] Song J Moon S Park DW Cho HJ Kim JY Park J. Biomarker combination and sofa score for the prediction of mortality in sepsis and septic shock: A prospective observational study according to the sepsis-3 definitions. Med (Baltimore) (2020) 99(22):e20495. doi: 10.1097/MD.0000000000020495 PMC12245219 32481464

[101] Song J Park DW Moon S Cho HJ Park JH Seok H. Diagnostic and prognostic value of interleukin-6, pentraxin 3, and procalcitonin levels among sepsis and septic shock patients: A prospective controlled study according to the sepsis-3 definitions. BMC Infect Dis (2019) 19(1):968. doi: 10.1186/s12879-019-4618-7 31718563 PMC6852730

[102] Friedland JS Porter JC Daryanani S Bland JM Screaton NJ Vesely MJ. Plasma proinflammatory cytokine concentrations, acute physiology and chronic health evaluation (Apache) iii scores and survival in patients in an intensive care unit. Crit Care Med (1996) 24(11):1775–81. doi: 10.1097/00003246-199611000-00003 8917024

[103] Hack CE De Groot ER Felt-Bersma RJ Nuijens JH Strack Van Schijndel RJ Eerenberg-Belmer AJ. Increased plasma levels of interleukin-6 in sepsis. Blood (1989) 74(5):1704–10. doi: 10.1182/blood.V74.5.1704.1704 2790194

[104] Jiang Y Jiang FQ Kong F An MM Jin BB Cao D. Inflammatory anemia-associated parameters are related to 28-day mortality in patients with sepsis admitted to the icu: A preliminary observational study. Ann Intensive Care (2019) 9(1):67. doi: 10.1186/s13613-019-0542-7 31183575 PMC6557959

[105] Mierzchala-Pasierb M Krzystek-Korpacka M Lesnik P Adamik B Placzkowska S Serek P. Interleukin-18 serum levels in sepsis: Correlation with disease severity and inflammatory markers. Cytokine (2019) 120:22–7. doi: 10.1016/j.cyto.2019.04.003 31003186

[106] Ridker PM MacFadyen JG Thuren T Libby P. Residual inflammatory risk associated with interleukin-18 and interleukin-6 after successful interleukin-1beta inhibition with canakinumab: Further rationale for the development of targeted anti-cytokine therapies for the treatment of atherothrombosis. Eur Heart J (2020) 41(23):2153–63. doi: 10.1093/eurheartj/ehz542 31504417

