# Supplementary figures and images for: A cytokine/PTX3 prognostic index as a predictor of mortality in sepsis
Source: Front Immunol. 2022 Sep 15;13:979232. doi: 10.3389/fimmu.2022.979232 (PMC9521428; doi:10.3389/fimmu.2022.979232)

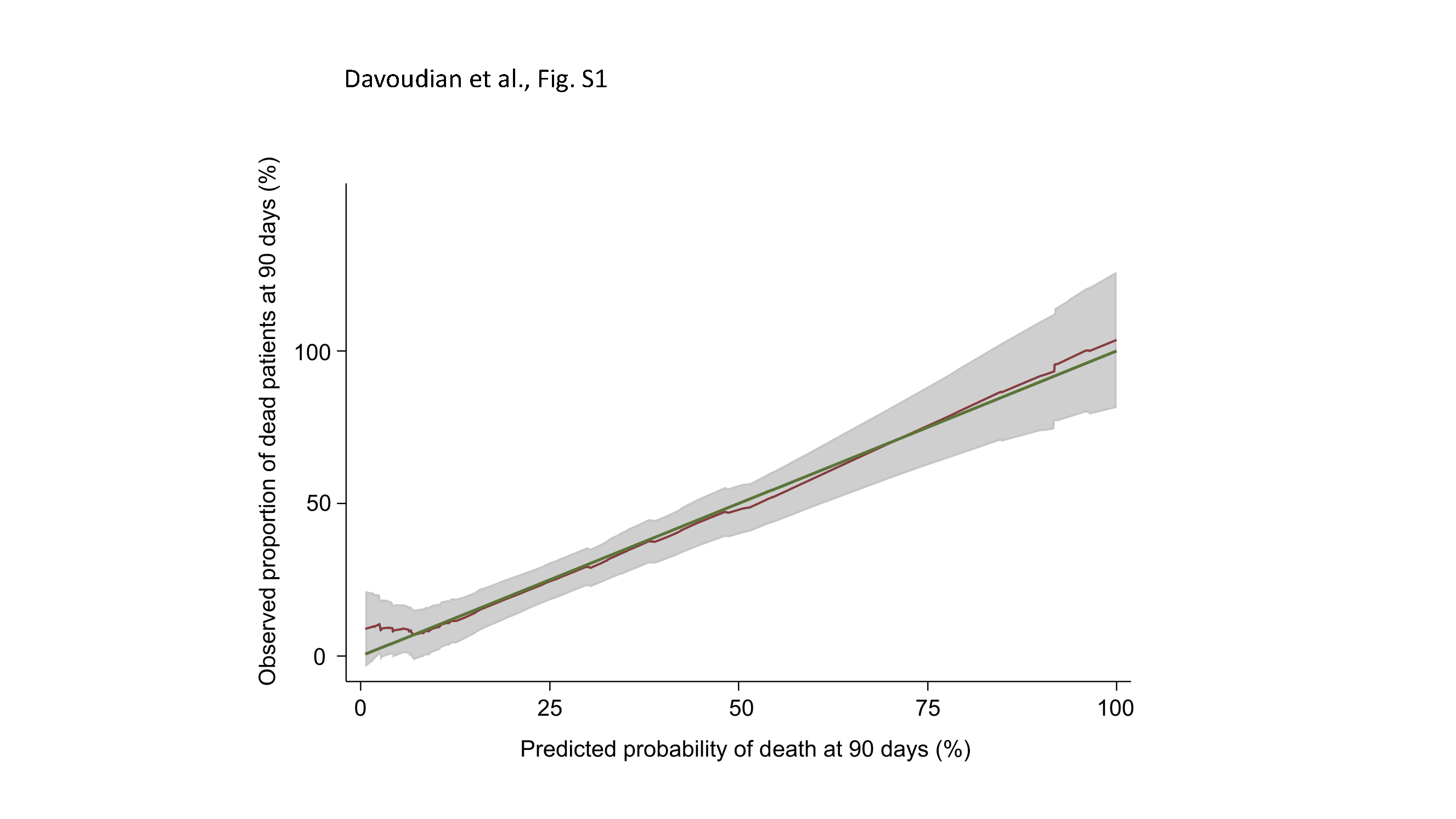

Supplement: Supplementary file 1 [file Image_1.tiff]

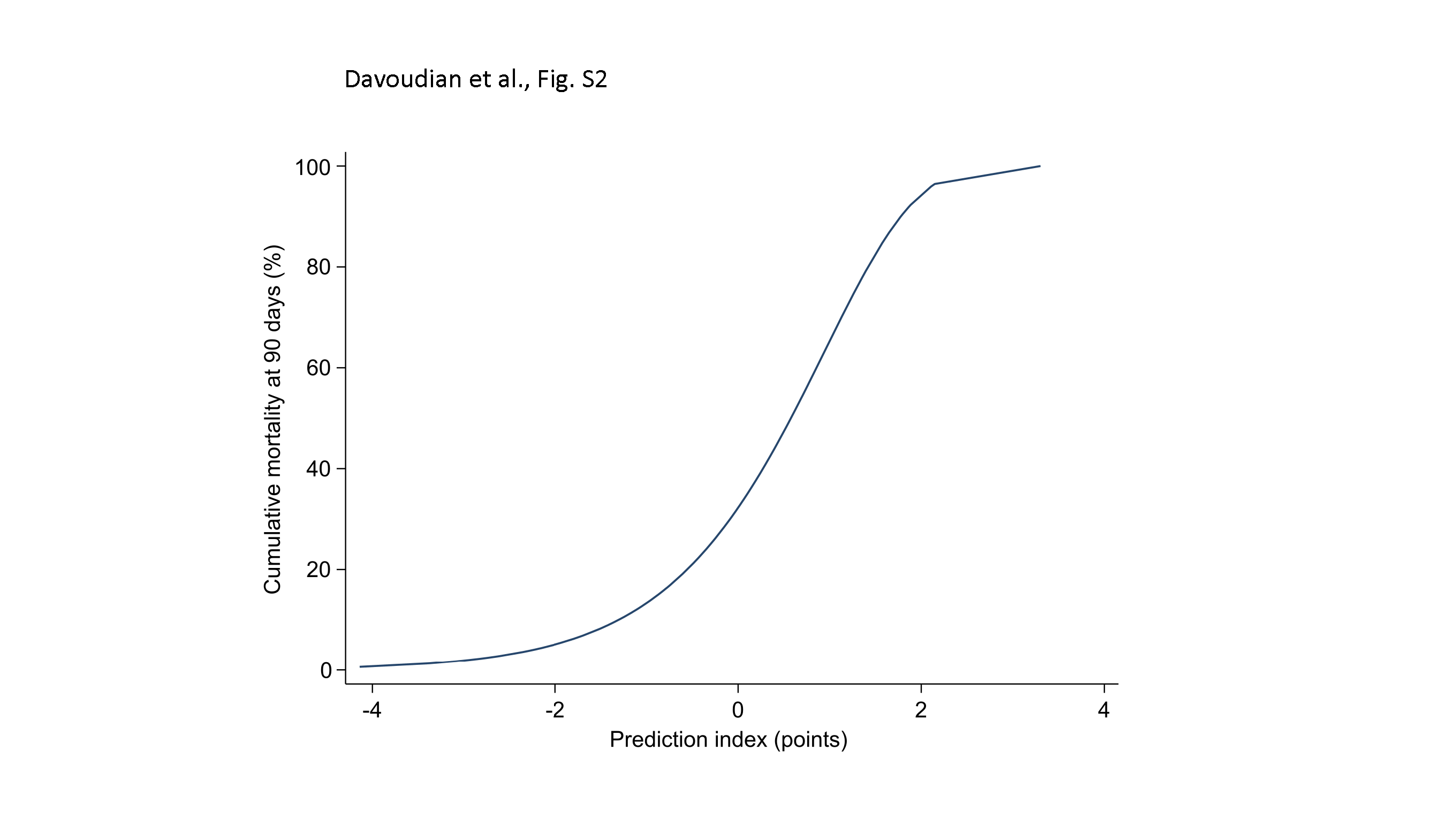

Supplement: Supplementary file 2 [file Image_2.tiff]
