# Supplementary material for: A cytokine/PTX3 prognostic index as a predictor of mortality in sepsis
Source: Front Immunol. 2022 Sep 15;13:979232. doi: 10.3389/fimmu.2022.979232 (PMC9521428; doi:10.3389/fimmu.2022.979232)
Supplement: Supplementary file 3 [file Table_1.docx]

Supplementary tables and figures

| ***Table S1. Univariable correlations of PTX3 and sIL-1R2 with clinical parameters in the Sepsis-3 population.*** | | | | | |
| --- | --- | --- | --- | --- | --- |
|  | **PTX3** | | **sIL-1R2** | |  |
| **Variables** | **Spearman r** | ***p* value** | **Spearman r** | ***p* value** | **n** |
| **SOFA score** | 0.44 | **<0.0001** | 0.35 | **<0.0001** | 135 |
| **qSOFA score** | 0.32 | **0.0001** | 0.11 | 0.19 | 135 |
| **WBC (10^3/mm^3)** | 0.10 | 0.24 | 0.17 | 0.03 | 134 |
| **Lymphocytes (10^3/mm^3)** | -0.28 | **0.0009** | -0.31 | **0.0003** | 134 |
| **Neutrophils (10^3/mm^3)** | 0.14 | 0.1 | 0.21 | **0.01** | 134 |
| **Platelets (10^3/mm^3)** | **-0.06** | 0.45 | -0.26 | **0.001** | 135 |
| **Lactate (mmol/L)** | 0.27 | **0.01** | 0.12 | 0.28 | 78 |
| **MAP (mmHg)** | **-0.31** | **0.0002** | -0.01 | 0.86 | 134 |
| **SBP (mmHg)** | -0.14 | 0.09 | -0.02 | 0.74 | 135 |
| **DBP (mmHg)** | -0.10 | 0.22 | -0.01 | 0.82 | 135 |
| **INR (sec)** | 0.22 | **0.01** | 0.16 | **0.06** | 124 |
| **Creatinine (mg/dL)** | 0.20 | **0.01** | 0.15 | **0.06** | 135 |
| **D-dimer (ng/ml)** | 0.30 | **0.008** | 0.34 | **0.002** | 72 |
| **Bilirubin (mg/dL)** | 0.12 | 0.18 | 0.30 | **0.0006** | 122 |
| **Urea (mg/dL)** | 0.15 | 0.07 | 0.22 | **0.01** | 134 |
| **PT (second)** | 0.21 | 0.15 | 0.18 | **0.03** | 131 |
| **Bicarbonate (mmol/L)** | -0.20 | 0.15 | -0.36 | **0.007** | 52 |
| **GCS** | -0.29 | **0.0004** | -0.06 | 0.46 | 135 |
| **PCT (ng/ml)** | 0.32 | **0.0002** | 0.43 | **<0.0001** | 124 |
| **CRP (mg/dL)** | 0.20 | **0.01** | 0.41 | **<0.0001** | 134 |
| **sIL-1R2 (ng/ml)** | 0.52 | **<0.0001** |  |  | 135 |
| **PTX3 (ng/ml)** |  |  | 0.52 | **<0.0001** | 135 |

*Spearman’s rank correlation test was used. Statistically significant values are in bold character. Number of patients is different due to missing data. Abbreviations: DBP, diastolic blood pressure; GCS, Glasgow coma scale; INR, international normalized ratio; MAP, mean arterial pressure; PCT, procalcitonin; PT, prothrombin time; SBP, systolic blood pressure; SOFA, Sequential Organ Failure Assessment; qSOFA, quick Sequential Organ Failure Assessment; WBC, white blood cells.*

| ***Table S2. Cytokine levels at day 1 and day 5 in non-sepsis, sepsis and septic shock patient.*** | | | |
| --- | --- | --- | --- |
| **Cytokines (pg/mL)** | **Day 1** | **Day 5** | ***p* value** |
| **Non-sepsis (n=23)** | | | |
| **IL-10** | 18.8 [7.9–38.7] | 7.3 [5.8–11.6] | **0.0002** |
| **IL-1β** | 0.8 [0.4–1.5] | 0.4 [0.3–0.6] | **<0.0001** |
| **IL-6** | 92.9 [51.7–270.0] | 22.6 [6.4–33.2] | **<0.0001** |
| **TNF-α** | 17.1 [11.5–36.4] | 11.7 [7.9–15.8] | **0.008** |
| **IL-18** | 262.6 [203.9–389.3] | 233.2 [191.1–358.7] | **0.02** |
| **IL-1ra** | 2846.0 [1538.0–7398.0] | 633.8 [437.7–1509.0] | **<0.0001** |
| **IL-8** | 16.5 [11.6–27.5] | 11.6 [9.2–18.8] | **0.02** |
| **Sepsis (n=62)** | | | |
| **IL-10** | 16.4 [8.0–41.2] | 7.3 [4.4–13.1] | **<0.0001** |
| **IL-1β** | 0.7 [0.4–1.1] | 0.4 [0.3–0.6] | **0.004** |
| **IL-6** | 133.8 [53.9–291.9] | 21.1 [11.6–70.7] | **<0.0001** |
| **TNF-α** | 27.4 [15.5–41.6] | 14.2 [10.5–19.5] | **<0.0001** |
| **IL-18** | 297.5 [225.8–582.4] | 312.9 [215.1–537.5] | 0.93 |
| **IL-1ra** | 4937.0 [1680.0–9869.0] | 793.9 [508.4–1828.0] | **<0.0001** |
| **IL-8** | 31.4 [20.1–70.4] | 15.3 [10.3–29.4] | **<0.0001** |
| **Septic shock (n=17)** | | | |
| **IL-10** | 32.8 [13.8–4332.0] | 14.2 [8.2–33.8] | **0.015** |
| **IL-1β** | 0.9 [0.6–7.7] | 0.5 [0.4–1.6] | 0.14 |
| **IL-6** | 557.6 [101.4–38637.0] | 47.9 [19.4–87.4] | **0.0005** |
| **TNF-α** | 46.4 [18.3–488.3] | 13.1 [7.6–23.8] | **0.0002** |
| **IL-18** | 311.8 [137.8–553.2] | 372.6 [237.9–554.5] | **0.03** |
| **IL-1ra** | 16058.0 [3390.0–135555.0] | 1137.0 [550.1–2025.0] | **<0.0001** |
| **IL-8** | 167.3 [32.2–2306.0] | 19.4 [14.8–72.5] | **0.002** |

*Cytokine values are reported as median pg/ml and [Q1-Q3] and refer only to those patients with available samples for both the time points. The Wilcoxon signed-ranks test was used for the comparisons. Statistically significant values are in bold character.*

| ***Table S3. Laboratory and clinical characteristics of Sepsis-3 population according to 90-days outcome.*** | | | | | |
| --- | --- | --- | --- | --- | --- |
| **Variables** | **Survivors** | **n** | **Non-Survivors** | **n** | ***p* value** |
| **Laboratory values** | | | | | |
| WBC (10^3/mm^3) | 11.7 [8.1-18.1] | 98 | 15.7 [9.2-20.6] | 36 | 0.11 |
| Lymphocytes (10^3/mm^3) | 0.7 [0.4-1.1] | 98 | 0.6 [0.4-1.0] | 36 | 0.45 |
| Platelets (10^3/mm^3) | 170 [124-256] | 99 | 209 [109-325] | 36 | 0.39 |
| INR (sec) | 1.3 [1.1-1.5] | 89 | 1.3 [1.2-1.6] | 35 | 0.15 |
| Fibrinogen (mg/dL) | 507 [414-706] | 50 | 542 [386-778] | 19 | 0.58 |
| Hematocrit (%) | 35.8 [31.8-39.9] | 99 | 33.8 [28.4-38.2] | 36 | 0.14 |
| Bilirubin(mg/dL) | 0.8 [0.6-1.2] | 92 | 1.0 [0.5-1.2] | 30 | 0.85 |
| Sodium (mmol/L) | 137 [133-140] | 99 | 137 [134-141] | 36 | 0.37 |
| Potassium (mmol/L) | 4.0 [3.6-4.5] | 98 | 4.4 [3.6-4.9] | 34 | 0.41 |
| Fasting blood sugar (mg/dL) | 122 [103-174] | 99 | 116 [96-178] | 36 | 0.53 |
| PT (second) | 1.3 [1.1-1.5] | 95 | 1.3 [1.2-1.6] | 36 | 0.11 |
| PTT (second) | 0.9 [0.9-1.1] | 95 | 1.0 [0.8-1.1] | 36 | 0.45 |
| PCT (ng/ml) | 5.6 [0.7-37.3] | 90 | 9.0 [0.6-22.8] | 34 | 0.85 |
| **Vital signs** | | | | | |
| Body mass index (kg/m²) | 25.7 [21.3-28.7] | 17 | 25.6 [22.3-25.6] | 6 | 0.74 |
| Respiratory rate (per minute) | 18 [16-20] | 78 | 18 [16-23] | 29 | 0.98 |
| Heart rate (bpm) | 100 [80-113] | 99 | 90 [77-112] | 36 | 0.28 |
| SBP (mmHg) | 107 [85-134] | 99 | 99 [88-114] | 36 | 0.27 |
| PaO2 (mmHg) | 72 [57-84] | 99 | 72 [63-86] | 36 | 0.36 |
| SpO2 (%) | 93 [89-96] | 99 | 93 [90.50-96] | 36 | 0.45 |
| PaO2/FiO2 | 2.9 [2.5-3.6] | 99 | 2.6 [1.8-3.4] | 36 | 0.1 |
| Bicarbonate (mmol/L) | 22.6 [19.7-27.1] | 37 | 20.6 [15.1-25.9] | 15 | 0.14 |
| PH value | 7.4 [7.4-7.5] | 49 | 7.5 [7.4-7.5] | 20 | 0.4 |

*Number of patients with available data is indicated for each variable. Data are reported as median and [Q1-Q3]; Mann-Whitney test was used for the comparisons. Abbreviations: FiO2, fraction of inspired oxygen; INR, international normalized ratio; PaO2, partial pressure of oxygen in arterial blood; PCT, procalcitonin; PT, prothrombin time; PTT, partial thromboplastin time; SBP, systolic blood pressure; SpO2, oxygen saturation. WBC, white blood cells.*

| **Table S4. Univariable Cox proportional hazard analysis of candidate predictors of 90-days mortality.** | | | |
| --- | --- | --- | --- |
| **Variable (n)** | **Hazard ratio** | **95% CI** | ***p* value** |
| **Age (years; n=135)** | 1.033 | 0.99‒1.06 | 0.059 |
| **Gender (n=135)** |  |  |  |
| **male** | ref. | ‒ | ‒ |
| **female** | 1.29 | 0.66‒2.52 | 0.452 |
| **WBC (10^6^/L; n=134)** | 1.01 | 0.98‒1.04 | 0.315 |
| **Lymphocytes (10^6^/L; n=134)** | 0.75 | 0.43‒1.33 | 0.338 |
| **Platelets (10^3^/μL; n=135)** | 1.00 | 0.99‒1.00 | 0.171 |
| **Creatinine (mg/dL; n=135)** | 1.12 | 0.99‒1.27 | 0.060 |
| **Haemoglobin (g/dL; n=135)** | 0.89 | 0.77‒1.02 | 0.116 |
| **Haematocrit (%; n=135)** | 0.97 | 0.93‒1.02 | 0.289 |
| **Potassium (mmol/L; n=132)** | 1.17 | 0.83‒1.65 | 0.362 |
| **Fasting blood sugar (mg/dL; n=135)** | 0.99 | 0.99‒1.00 | 0.691 |
| **PT (s; n=131)** | 0.99 | 0.68‒1.46 | 0.996 |
| **PTT (sec; n=131)** | 1.59 | 0.76‒3.31 | 0.213 |
| **Body temperature (C°; n=132)** | 0.87 | 0.75‒1.01 | 0.076 |
| **Heart rate (pulse/min; n=135)** | 0.99 | 0.98‒1.00 | 0.492 |
| **SBP (mmHg; n=135)** | 0.99 | 0.98‒1.00 | 0.286 |
| **DBP (mmHg; n=135)** | 0.98 | 0.96‒1.00 | 0.085 |
| **PaO_2_ (mmHg; n=135)** | 1.00 | 0.98‒1.02 | 0.484 |
| **SpO_2_ (%; n=135)** | 0.99 | 0.97‒1.01 | 0.644 |
| **Comorbidities** |  |  |  |
| **Cardiovascular diseases (n=135)** | 1.63 | 0.85‒3.15 | 0.139 |
| **Hypertension (n=135)** | 1.10 | 0.56‒2.18 | 0.768 |
| **Malignancy (n=135)** | 1.29 | 0.66‒2.53 | 0.448 |
| **Neurological diseases (n=135)** | 0.72 | 0.30‒1.73 | 0.463 |
| **Type 2 diabetes (n=135)** | 1.02 | 0.44‒2.33 | 0.957 |
| **Chronic obstructive pulmonary disease (n=135)** | 1.00 | 0.45‒2.21 | 0.985 |
| **Chronic kidney disease (n=135)** | 1.73 | 0.81‒3.68 | 0.153 |
| **Other comorbidities (n=135)** | 1.04 | 0.45‒2.37 | 0.924 |
| **Biomarkers of inflammation (n=135)** | | | |
| **TNF-α (pg/mL)** | 1.61 | 0.92‒2.81 | 0.089 |
| **IL-18 (pg/mL)** | 1.83 | 0.62‒5.38 | 0.270 |

*Levels of TNF-α and IL-18 were log10 transformed before statistical analysis. Abbreviations:* *CI, confidence interval; DBP, diastolic blood pressure; PaO2, partial pressure of oxygen in arterial blood;PCT, procalcitonin; PT, prothrombin time; PTT, partial thromboplastin time; SBP, systolic blood pressure; SOFA, Sequential Organ Failure Assessment; SpO2, oxygen saturation; qSOFA, quick Sequential Organ Failure Assessment; WBC, white blood cells.*

**Legend to Supplementary Figures**

**Figure S1. *Calibration plot of the observed proportion against expected probability of death at 90-days.*** The line in red represents the calibration plot while in gray is the 95% CI; the green line represents perfect calibration (i.e. slope = 1 and intercept = 0).

***Figure S2. Nomogram plot.*** The plot represents the association of the individual 90-days risk of death with each possible prognostic index derived from applying the multivariable model.

**
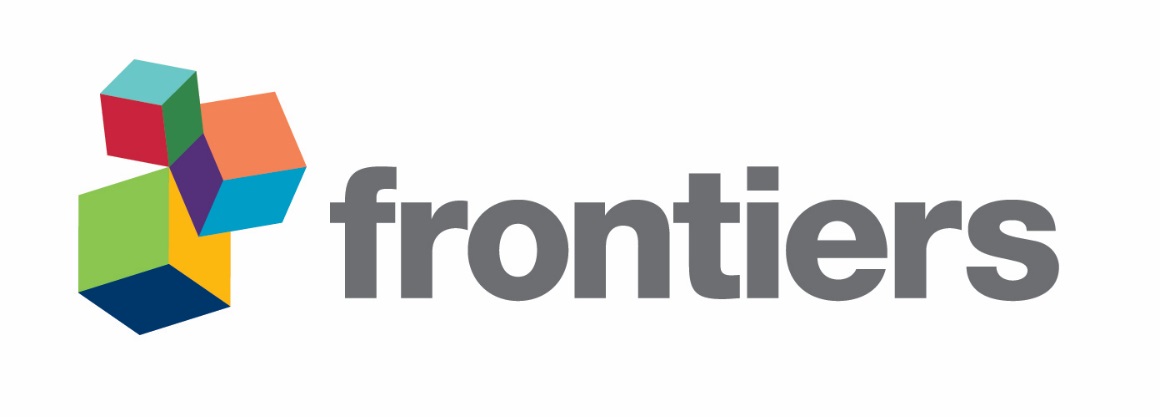
**
